# Dendritic osmosensors modulate activity-induced calcium influx in oxytocinergic magnocellular neurons of the mouse PVN

**DOI:** 10.7554/eLife.63486

**Published:** 2021-07-12

**Authors:** Wanhui Sheng, Scott W Harden, Yalun Tan, Eric G Krause, Charles J Frazier

**Affiliations:** 1 Department of Pharmacodynamics, College of Pharmacy, University of Florida Gainesville United States; 2 Department of Anesthesiology, School of Medicine, Stanford University Stanford United States; 3 Center for Integrative Cardiovascular and Metabolic Diseases, University of Florida Gainesville United States; 4 Evelyn F. and William L. McKnight Brain Institute, University of Florida Gainesville United States; Max Planck Florida Institute for Neuroscience United States; Stanford University School of Medicine United States

**Keywords:** oxytocin, hypothalamus, magnocellular neuron, PVN, dendrite, electrophysiology, Mouse

## Abstract

Hypothalamic oxytocinergic magnocellular neurons have a fascinating ability to release peptide from both their axon terminals and from their dendrites. Existing data indicates that the relationship between somatic activity and dendritic release is not constant, but the mechanisms through which this relationship can be modulated are not completely understood. Here, we use a combination of electrical and optical recording techniques to quantify activity-induced calcium influx in proximal vs. distal dendrites of oxytocinergic magnocellular neurons located in the paraventricular nucleus of the hypothalamus (OT-MCNs). Results reveal that the dendrites of OT-MCNs are weak conductors of somatic voltage changes; however, activity-induced dendritic calcium influx can be robustly regulated by both osmosensitive and non-osmosensitive ion channels located along the dendritic membrane. Overall, this study reveals that dendritic conductivity is a dynamic and endogenously regulated feature of OT-MCNs that is likely to have substantial functional impact on central oxytocin release.

## eLife digest

Oxytocin is often referred to as a ‘love hormone’ because it can be released during activities such as hugging, snuggling, or sex. Reality, of course, can be a bit more complicated. In the brain, oxytocin can have powerful and diverse effects on mood, stress, anxiety, and social interactions. In the body it helps regulate fluid balance, promotes contractions during childbirth, and stimulates the letdown of milk during breastfeeding.

Much of the oxytocin produced in both humans and rodents comes from oxytocin-synthetizing magnocellular neurons located in an area of the brain called the hypothalamus. These very specialized neurons have separate, but overlapping, mechanisms for releasing oxytocin into the brain and into the rest of the body. This means that while certain signals cause the neurons to release oxytocin into the body and the brain at the same time, others can cause them to release the hormone preferentially into the body or the brain.

Sheng et al. wanted to better understand how these different release mechanisms work, and, in particular, to learn more about how release of oxytocin into the brain is regulated. This is important, because when oxytocin is given as a medicine, much of it fails to reach the brain.

A lot of the oxytocin that acts in the brain is released from a specific part of the oxytocin-synthesizing magnocellular neurons called the dendrites. When these neurons are stimulated, calcium enters the dendrites, triggering the release of oxytocin directly into the brain. Sheng et al. used electrical and optical tools on brain tissue extracted from mice to measure how different signals change the amount of calcium that enters the dendrites of oxytocin-synthesizing magnocellular neurons in response to a consistent stimulus.

The results showed that increasing the osmolarity, the amount of water-soluble particles that cannot spontaneously cross the cell membrane, in the liquid surrounding the neurons reduced the amount of calcium that flowed into the dendrites during stimulation. Meanwhile, decreasing osmolarity had the opposite effect. Sheng et al. also found that the influx of calcium induced by stimulating the neurons can be strongly regulated by activating receptors in the dendrites that detect a common molecule in the brain called GABA. This occurs even absent a change in osmolarity.

These results shed light on some of the physiological processes that control the release of oxytocin into the brain. Understanding these processes is a necessary step towards developing new drugs intended to regulate levels of oxytocin in the brain. Such drugs could be useful in the treatment of several types of mental health disorders.

## Introduction

Oxytocin (OT) is a nine amino acid peptide synthesized almost exclusively in hypothalamic neurons of the supraoptic and paraventricular nucleus (SON, PVN). Despite the highly localized nature of OT synthesizing neurons, OT receptors (OTRs) are distributed widely throughout the CNS. Significant evidence indicates an important modulatory role for central oxytocinergic signaling in a wide variety of processes including fear conditioning, stress responding, anxiety-related behaviors, maternal behavior, sexual behavior, and social recognition (e.g. see Bosch et al., 2005; Knobloch et al., 2012; Jurek et al., 2015; Veening et al., 2015; Neumann and Slattery, 2016; Lin et al., 2018; Tan et al., 2019; Valtcheva and Froemke, 2019; Winter and Jurek, 2019). Numerous studies have demonstrated effects of OTR agonists and antagonists delivered intracerebrally or intraventricularly on social, stress, and anxiety-related behaviors, while conversely, animal models with genetic disruptions in OT signaling systems exhibit a range of social and behavioral deficits (e.g. see Jin et al., 2007; Young, 2007; Lee et al., 2008; Higashida et al., 2010; Pobbe et al., 2012; Kent et al., 2013; Morales-Rivera et al., 2014; Peters et al., 2014; Burkett et al., 2016; Caldwell et al., 2017; Lee et al., 2018). Collectively, these types of data implicate the central OT signaling system as a promising therapeutic target for a variety of conditions impacting mental health. However, effective therapeutic delivery of exogenous OTR agonists into the CNS of humans remains difficult (Ermisch et al., 1985; McEwen, 2004; Veening and Olivier, 2013; Leng and Ludwig, 2016; Quintana and Woolley, 2016; De Cagna et al., 2019), and thus a more detailed mechanistic understanding of how the brain naturally regulates release of endogenous OT may facilitate development of new approaches for therapeutic manipulation of central OT signaling.

The question of exactly how, and from where, endogenous OT is released to act on both hypothalamic and extrahypothalamic OTRs in the CNS is a complex one. It is complicated by the fact that there are both magnocellular and parvocellular hypothalamic OT synthesizing neurons (OT-MCNs, OT-PCNs), and by the fact that OT-MCNs can release peptide both from their axon terminals and from their dendrites. The current study focuses on dendritic physiology of PVN OT-MCNs because (1) OT-MCNs substantially outnumber OT-PCNs (Althammer and Grinevich, 2017), (2) most axons of OT-MCNs project through the median eminence to the posterior pituitary where activity (action potential) dependent release into the vasculature increases peripheral (and not central) OT concentration (Guzek, 1987; Robinson et al., 1989; Falke, 1991), (3) a large portion of OT available for release into the CNS exists in dendritic rather than axonal vesicles that are subject to activity, and calcium, dependent exocytosis (Pow and Morris, 1989; Ludwig et al., 2002; Ludwig and Leng, 2006), (4) such exocytosis supports functionally important peptide mediated paracrine signaling within the hypothalamus (Son et al., 2013; Smith et al., 2015; Pati et al., 2020), and (5) likely also drives volume transmission to increase functional activation of OTRs in a variety of extrahypothalamic cortical and limbic areas (Veening et al., 2010; Fuxe et al., 2012; Ludwig and Stern, 2015; Brown et al., 2020). Indeed, this mechanism seems likely to work in concert with limited/targeted release from centrally projecting axon collaterals of OT neurons, as has been effectively demonstrated in several extrahypothalamic areas to date (Knobloch et al., 2012; Eliava et al., 2016; Oettl et al., 2016).

In the current study, we use a combination of electrophysiological and subcellular optical recording techniques to evaluate dendritic physiology of OT-MCNs. Somatic activity was induced with a reproducible train of action potential-like voltage pulses delivered to the soma, and calcium influx induced by this somatic activity was quantified using high frequency two-photon line scans across the proximal and distal dendrite. The results reveal that the dendrites of OT-MCNs are weak conductors of somatic voltage changes. We further report that acute hyperosmotic challenge preferentially reduces activity-induced calcium influx in distal vs. proximal OT-MCN dendrites, while acute hypoosmotic challenge increases it. Extensive control experiments indicate these effects are likely mediated by modulation of a previously unidentified osmosensitive channel expressed along the dendritic membrane. Finally, we report that that activity-induced calcium influx in the distal dendrites of OT-MCNs is also preferentially and robustly inhibited, absent any change in osmolarity, by activation of dendritic GABA_A_ receptors. Collectively, these results significantly increase our understanding of the mechanisms through which OT-MCNs are likely to dynamically regulate the relationship between somatic activity and calcium-dependent dendritic release of OT into the CNS.

## Results

### Identification of OT-MCNs and PCNs in vitro

**Figure 1.**
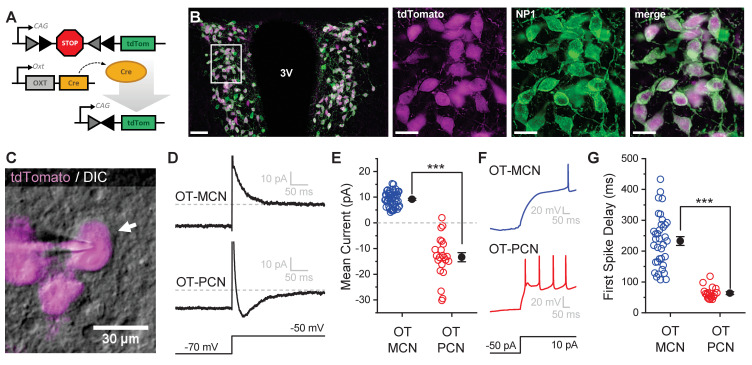
An oxytocin (OT) reporter mouse line facilitates selective targeting of OT neurons for in vitro experiments. (**A**) Fluorophore (tdTomato) is selectively expressed in OT neurons of OT-Cre mice using a stop-floxed tdTomato construct. (**B**) Immunohistochemistry demonstrates colocalization of tdTomato and NP1 in PVN neurons. See Results for additional details. (**C**) Endogenous tdTomato facilitates selective targeting of OT neurons in acute brain slices for whole-cell patch clamp recording. (**D**) Magnocellular neurons (OT-MCNs, top panel) can be distinguished from parvocellular neurons (OT-PCNs, bottom panel) by the presence of a transient outward current (*I_A_* current) following a voltage-clamp step from −70 mV to −50 mV. Outward current in OT-MCNs is quantified over the first 100 msec after the step to −50 mV, relative to the steady state current at that voltage which is indicated by the dashed line. By contrast, OT-PCNs often reveal a transient inward current in response to the same stimulus (quantified with the same strategy, bottom panel). (**E**) Mean current response to the voltage step as presented in panel D segregates MCNs (demonstrating a transient outward current, I_A_) from PCNs (with no or inward transient current, n=51, 22, t = 12.46, p<0.001, unpaired two-sample t-test). (**F**) The *I_A_* current in MCNs produces a delay to first spike which can be readily observed in response to a suprathreshold depolarizing step in current clamp (top panel). (**G**) Time to first spike, as observed in response to a 40 pA current injection that immediately followed a hyperpolarizing prepulse, is significantly longer in OT-MCNs vs. OT-PCNs (n=36, 19, t = 11.41, p<0.001, unpaired two-sample t-test). Scale bars: B left panel 100 µm, B right panels 20 µm, C 30 µm. Error bars represent mean ± SEM. Figure 1—source data 1.Excel file containing data for panels E and G.

All experiments for this study were performed in an OT-reporter mouse line designed to selectively expresses a red fluorescent protein (tdTomato) in oxytocinergic neurons (Figure 1A, see Materials and methods). In order to validate specificity and selectivity of this reporter in the PVN, we used immunohistochemical techniques to evaluate co-expression of tdTomato and neurophysin 1 (NP1, an OT carrier protein found in oxytocinergic neurons, Figure 1B). Overall, we found that a strong majority of tdTomato expressing neurons in the PVN were immunoreactive for NP1 (92.8 ± 0.8% across all animals tested, 93.2 ± 1.0% in males, and 92.4 ± 0.5% in females, n = four animals total, two male, two female, 2838 total tdTomato-positive PVN neurons evaluated, 1352 from males, and 1486 from females). Based on these data, we used a combination of IR-DIC and epifluorescence microscopy (see Figure 1C and Materials and methods) to efficiently target PVN OT neurons for whole-cell patch clamp recording. Once patched, PVN OT neurons were categorized as either magnocellular or parvocellular based on the presence or absence (respectively) of a transient outwardly rectifying potassium current, I_A_ (Luther and Tasker, 2000). This current was readily apparent as an outward current in voltage clamp observed in the first 100 msec after stepping from −70 mV to −50 mV (Figure 1D–E). It also contributed to a clear delay to first action potential observed in current clamp in response to a suprathreshold current injection (Figure 1F–G). By contrast, OT-PCNs not only lacked I_A_, but often also displayed a small inward current after stepping from −70 mV to −50 mV in voltage clamp (Figure 1D, bottom trace). This current often produced a low threshold spike that was apparent in current clamp (Figure 1F, bottom trace), and contributed to the short delay to first spike observed in OT-PCNs (Figure 1G). We noted that OT-MCNs had significantly higher somatic input resistance than OT-PCNs (1836.87 ± 107.84 MΩ vs. 854.81 ± 73.92 MΩ, respectively, n=62, 22, t = 7.5, p<0.0001, unpaired two-sample t-test), while whole-cell capacitance at −70 mV did not significantly differ (Cm: 31.1 ± 1.4 pF vs. 29.82 ± 1.8 pF, n=62, 22, t = 0.5, p = 0.621, unpaired two-sample t-test). Importantly, none of the core intrinsic electrophysiological features of OT-MCNs reported here varied by sex (See Figure 1—figure supplement 1 and legend).

**Figure 1—figure supplement 1.**
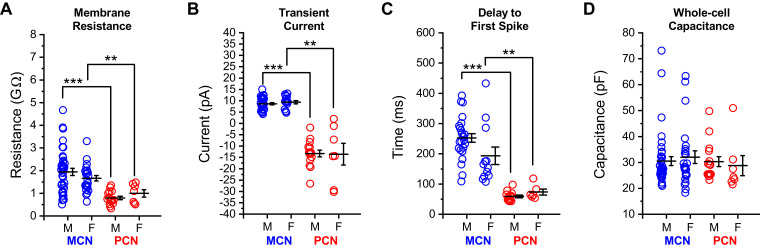
Intrinsic properties of OT PVN neurons do not differ by sex. Core intrinsic properties of OT-MCNs (blue) and OT-PCNs (red) are separated by both sex and cell type and illustrated in panels A-D. A two-way ANOVA was run on data in each panel with sex and cell type as factors. (**A–C**) In these panels, illustrating membrane resistance, transient current amplitude, and delay to first spike, respectively, a two-way ANOVA revealed that there was no significant effect of sex (Rm: F_1,83_ = 0.04, p = 0.84, transient current: F_1,66_ = 0.02, p = 0.89, delay to first spike: F_1,54_ = 0.05, p = 0.83), there was a main effect of cell type (Rm: F_1,83_ = 21.2, p = 1.54 x 10^−5^, transient current: F_1,66_ = 230.3, p = 1 x 10^−22^, delay to first spike: F_1,54_ = 33.95, p = 3.83 x 10^−7^), and there was no significant interaction between sex and cell type (Rm: F_1,83_ = 1.496, p = 0.225, transient current: F_1,66_ = 0.12, p = 0.73, delay to first spike: F_1,54_ = 1.22, p = 0.274). Based on these results, we performed an unpaired two-sample t-test on selected data in these panels using data within each sex, separated by cell type. The results of these analyses, highlighted with brackets in panels A-C, indicate that in OT-MCNs vs. OT-PCNs, both sexes had significantly larger input resistance, displayed an outward rather than an inward transient current in voltage clamp, and had a longer delay to first spike in current clamp (* = p < 0.05, ** = p< 0.01, and *** = p < 0.001). (**D**) In contrast, a two-way ANOVA run on measurements of whole-cell capacitance, with both sex and cell type as factors, revealed no significant effect of either sex or cell type (sex: F_1,83_ = 5.7 x 10–5, p = 0.99, cell type: F_1,83_ = 0.392, p = 0.53). Overall, based on results presented in panels A-D, data were combined across sex and separated by cell type for analyses presented in Figure 1, and in related sections of the results. N values for cells and animals represented in all panels above are as follows: Panels A and D: OT-MCN male: 38, 15, OT-MCN female: 24, 12, OT-PCN male: 15, 3, OT-PCN female: 7, 6, Panel B: OT-MCN male: 30, 11, OT-MCN female: 15, 7, OT-PCN male: 15, 3, OT-PCN female: 7, 6, and Panel C: OT-MCN male: 24, 9, OT-MCN female: 12, 6, OT-PCN male: 13, 3, OT-PCN female: 6, 5. Figure 1—figure supplement 1—source data 1.Excel file containing data for panels A-D.

### Acute hyperosmotic challenge increases basal firing rate of OT-MCNs in vitro

**Figure 2.**
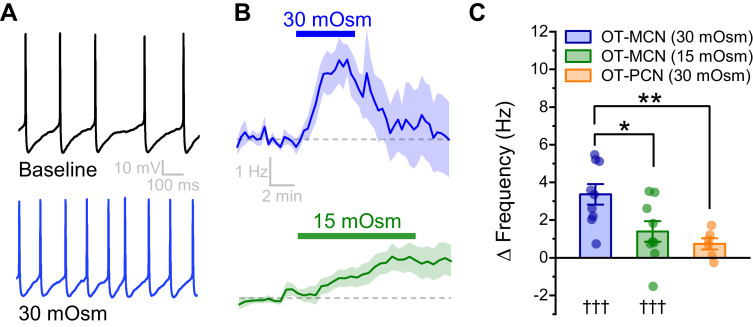
An acute hyperosmotic stimulus increases OT-MCN activity. (**A**) Spontaneous firing in a representative OT-MCN before (top) and after (bottom) exposure to MT (30 mOsm). (**B**) Change in spontaneous action potential frequency over time in response to +30 mOsm (n=9, top) and +15 mOsm (n=9, bottom). (**C**) Both 15 and 30 mOsm stimuli caused a significant increase in action potential frequency in OT-MCNs (denoted by †††, p<0.001, one-sample t-tests), but response to 30 mOsm stimulus was significantly larger (denoted by * p< 0.01, unpaired two-sample t-test, blue vs. green bar). By contrast, the same 30 mOsm stimulus did not produce a significant increase in firing rate in OT-PCNs (orange bar, p = 0.06, one-sample t-test), and these results were significantly different that those observed in OT-MCNs (** denotes p = 0.003, unpaired two-sample t-test). Figure 2—source data 1.Excel file containing data for panel C.

In order to directly evaluate the osmosensitivity of PVN OT-MCNs in vitro, we used whole-cell current clamp recordings, in combination with bath application of mannitol (MT, an inert osmolyte), to evaluate the effect of acute hyperosmotic challenge on firing frequency. We found that bath application of 30 mOsm MT for 5 min (in the presence of glutamate and GABA receptor antagonists, see Materials and methods) increased basal firing rate of OT-MCNs by 3.37 ± 0.55 Hz (n = 9, t = 6.2, p = 0.0002, Figure 2A–B.) A smaller but longer lasting hyperosmotic stimulus (15 mOsm MT for 10 mins) increased basal firing rate by 1.39 ± 0.55 Hz (n=9, t = 2.6, p = 0.03, Figure 2B–C), and was chosen as a standard hyperosmotic stimulus throughout the rest of the study. This effect of MT is cell type specific, as 30 mOsm MT failed to produce a similar effect in PVN OT-PCNs (ΔFreq: 0.7 ± 0.3 Hz, t = 2.49, p = 0.06, t = 3.58, p = 0.005 vs. effect of 30 mOsm MT observed in OT-MCNs, Figure 2C). Although additional synaptic input from osmosensitive circumventricular organs might further enhance OT-MCN activity in vivo during times of increased osmolarity, the present results demonstrate PVT OT-MCNs are directly osmosensitive and highlight that a primary effect of acute hyperosmotic challenge, when observed somatically, is excitatory.

### OT-MCN dendrites are weak conductors of somatic voltage changes

**Figure 3.**
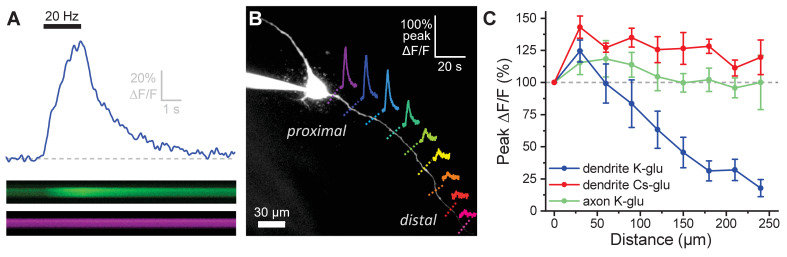
Activity-induced dendritic calcium influx decreases with distance from the soma in OT-MCNs. (**A**) Blue trace illustrates calcium response observed in an OT-MCN dendrite as produced by a 2 s train of action potential-like voltage steps delivered to the soma at 20 Hz (black bar). The calcium signal was calculated using emissions from a calcium sensitive and calcium insensitive indicator (Fluo-5F and Alexa Fluor 594, respectively) obtained by performing a two-photon line scan across the dendrite during somatic stimulation. The bottom panels illustrate line scan data (top: Fluo-5F, bottom: Alexa Flour 594), with time on the horizontal axis and space across the dendrite on the vertical axis. See Materials and methods for details. (**B**) Two-photon Z-series projection of a representative OT-MCN. Colored dashed lines indicate position of line scans performed in this cell during somatic stimulation as in panel A. Solid lines illustrate calcium response, calculated as in panel A, observed at each location. Scale bar in the top right applies to all traces. (**C**) Illustrates the peak of the calcium response as a percentage of baseline (most proximal response) plotted against distance from the soma. Data were obtained from OT-MCN dendrites using either a K-gluconate or Cs-gluconate-based internal solution (blue vs. green traces, respectively), and from the axon of OT-MCNs (red trace). Overall, these data reveal significant distance-dependent loss of activity-induced calcium influx only in OT-MCN dendrites, and only in cells that were not filled with a Cs-gluconate-based internal solution. Figure 3—source data 1.Excel file containing data for panel C.

It is clear from prior literature that dendritic release of OT from MCNs is calcium dependent, and it is often presumed that somatic action potentials lead to dendritic calcium influx in a way that promotes dendritic release of peptide (Di Scala-Guenot et al., 1987; Fisher and Bourque, 1996; Tobin et al., 2012). That said, the relationship between somatic activity and dendritic release of OT is likely not constant (see Discussion), and key aspects of dendritic physiology potentially impacting this relationship have not been directly examined before. For these reasons, we used a combination of electrophysiological and optical techniques to quantify activity-induced calcium influx along the length of OT-MCN dendrites as observed in response to a two-second train of action potential like voltage steps delivered to the soma at 20 Hz (Figure 3A, calcium indicator delivered via the patch pipette, see Materials and methods). This approach revealed that activity-induced calcium influx drops rapidly with increasing distance from the soma (main effect of distance, F_1.6,,6.4_ = 15.0, p = 0.005, one-way repeated measures ANOVA, Figure 3B, Figure 3C, blue trace), suggesting that under basal conditions OT-MCN dendrites lack the ability to actively propagate somatic voltage changes to distal locations, and likely also have a relatively low membrane resistance. However, similar data might be expected if calcium indicators had failed to perfuse into the distal dendrites, or if distal dendrites expressed substantially fewer voltage-gated calcium channels. To evaluate both possibilities simultaneously, we repeated the experiment using a cesium gluconate internal solution (see Materials and methods) to block cesium-sensitive leak channels and thereby increase dendritic membrane resistance. Notably, in these conditions, we observed an overall increase in activity-induced calcium influx (main effect of internal, F_1,9_ = 30.55, p = 3.7 x 10^−4^, two-way repeated measures ANOVA, Figure 3C, red trace), with post-hoc tests indicating significantly increased response at distances > 60 µm from the soma. These results indicate that functional calcium indictor was present in the distal dendrites, and that voltage-gated calcium channels are still robustly expressed at distal locations. As such, they also substantially reinforce the conclusion that loss of activity-induced calcium influx at increasing distance from the soma, as observed when using a more physiological K-gluconate-based internal solution, was produced by loss of current through open channels along the dendritic membrane.

**Figure 3—figure supplement 1.**
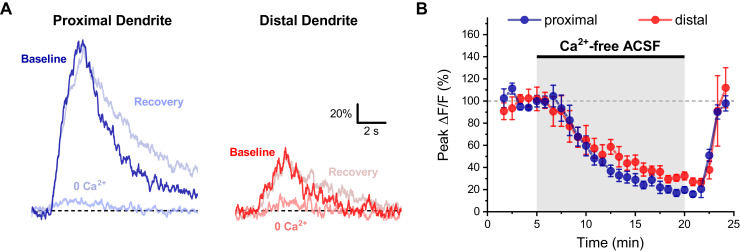
Activity-induced dendritic calcium influx in OT-MCN dendrites requires external calcium. (**A**) Representative traces of activity-induced calcium influx measured at proximal and distal dendritic locations (blue traces, red traces, respectively) during baseline conditions, after 15 min of bath application of calcium-free ACSF, and after an additional 5 min recovery period. (**B**) Summary data indicates a significant reduction of activity-induced calcium influx in both proximal and distal dendrites, followed by a rapid recovery (see text of results for further details). Figure 3—figure supplement 1—source data 1.Excel file containing data for panel B.

**Figure 4.**
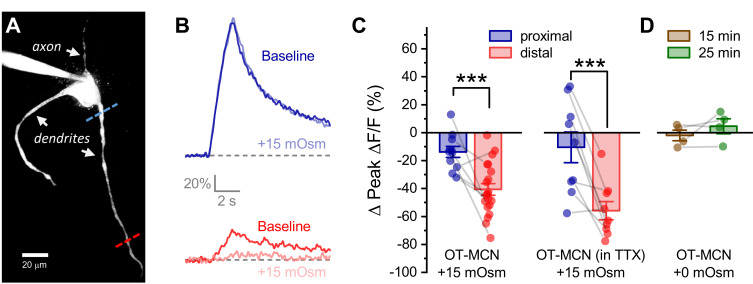
Acute hyperosmotic challenge produces a distance-dependent reduction in activity-induced calcium influx, as observed in OT-MCN dendrites. (**A**) A two-photon Z-series projection image of a representative OT-MCN indicating proximal (blue) and distal (red) calcium imaging locations. (**B**) Activity-induced calcium response observed before and during exposure to a 15 mOsm stimulus at proximal and distal sites (top, bottom of panel respectively, with baseline responses color coded to match line scan positions indicated in panel A). (**C**) *Left panel:* Across multiple cells tested, the 15 mOsm stimulus produced a small inhibitory effect on activity-induced calcium influx as observed in the proximal dendrites (−14.3 ± 3.37%, n = 13, t = −4.2, p = 0.001, one-sample t-test), and a significantly larger inhibitory effect as observed in the distal dendrites (Left panel, *** p = 1.1 x 10^−4^, n=13, 20, for proximal and distal dendrites, respectively. See text of results for further details). Note an unpaired two-sample t-test was used for this analysis because some cells had data only from the proximal or the distal location. In cases were both proximal and distal data is available within a cell, corresponding points are connected by grey lines. If analysis is restricted to paired data only the difference between the effect observed in proximal and distal dendrites is still statistically significant (proximal: −15.27 ± 4.66%, distal: −45.67 ± 6.39%, n=9, t = 3.51, p = 0.008, paired two-sample t-test). *Right panel:* Although raw calcium influx was reduced in cells pretreated with 1 µM TTX, a 15 mOsm stimulus continued to preferentially inhibit activity-induced calcium influx as observed in the distal dendrites (right panel, *** p = 0.003, n=9,9 for distal and proximal dendrites, respectively, paired two-sample t-test). (**D**) Absent hyperosmotic stimulus, activity-induced calcium influx measured as in panels A-C is stable in distal dendrites over a 25-min recording period (sufficient time to change bath conditions in earlier experiments, n=four for distal dendrites, see text of results for further details). Figure 4—source data 1.Excel file containing source data for panels C-D.

Next, we repeated the experiment for a third time, using a K-gluconate-based internal solution, to evaluate activity-induced calcium influx along the length of OT-MCN axons. Axons were distinguished from dendrites based primarily on their smaller initial diameter as observed with 2P epifluorescence microscopy (e.g. Figure 4A). However, consistent with prior reports (Hatton, 1990; Stern and Armstrong, 1998), we also noted that axons often (but not always) branch off a primary dendrite very close to the soma. The results of this experiment indicated that OT-MCN axons, unlike dendrites, reliably and actively propagate somatic voltage changes in cells filled with potassium gluconate (Figure 3C, green trace). Specifically, a two-way repeated measures ANOVA revealed a main effect of structure on activity-induced calcium influx (axon vs. dendrite, both recorded using a K-gluconate-based internal solution, F_1,4_ = 9.17, p = 0.04), while post-hoc tests revealed that significantly less loss of response was observed in axons compared to dendrites at distances > 90 µm from the soma. Interestingly, these data also highlight expression of voltage-gated calcium channels along the length of OT-MCN axons.

**Figure 4—figure supplement 1.**
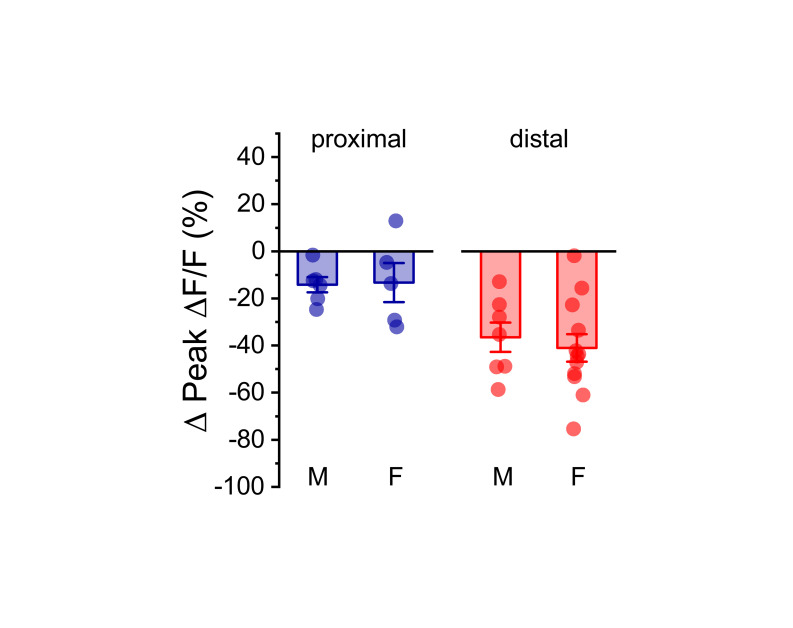
Effect of acute hyperosmotic challenge on activity-induced calcium influx is similar in males and females. Summary data, separated by sex (M/F), illustrating the effect of acute hyperosmotic challenge on activity-induced calcium influx in both proximal (blue) and distal (red) dendrites of OT-MCNs. A two-way ANOVA run on these data, with sex and dendritic location as factors, revealed no main effect of sex (F_1,29_ = 0.08, p = 0.78), a significant effect of dendritic location (F_1,29_=14.1, p = 8.8 x 10^−4^), and no significant interaction between sex and location (F_1,29_ = 0.17, p = 0.68). As such, these data were combined across sex, separated by location, and presented in Figure 4C, left panel. For proximal data, n = 6 cells, 3 animals, and 5 cells, 5 animals for males and females, respectively. For distal data, n = 7 cells, 6 animals, and 12 cells, 7 animals for males and females, respectively. Figure 4—figure supplement 1—source data 1.Excel file containing source data.

**Figure 4—figure supplement 2.**
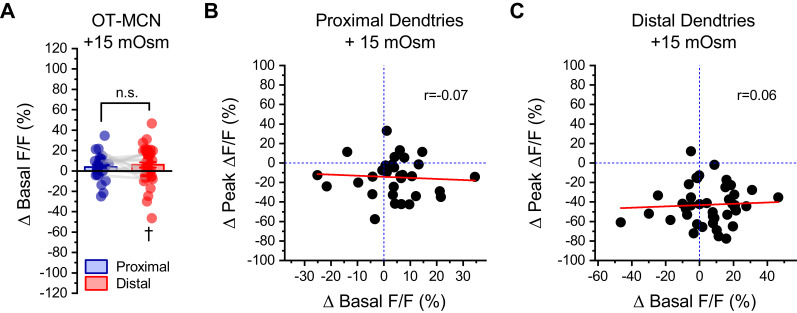
Changes in basal calcium observed during acute hyperosmotic challenge are not predictive of changes in activity-induced calcium influx, as observed in either proximal or distal dendrites of OT-MCNs. (**A**) Across multiple experiments that involved acute hyperosmotic challenge with +15 mOsm MT, basal calcium (as indicated by resting F/F) increased by 4.0 ± 2.1% in the proximal dendrites of OT-MCNs (n=32, t = 1.8, p = 0.07, one-sample t-test), and by 6.1 ± 2.6% in the distal dendrites (n=43, t = 2.3, p = 0.02 †, one-sample t-test against null hypothesis that mean = 0). An unpaired two-sample t-test indicates that these changes were not significantly different from one another (t = −0.69, p = 0.49). (**B–C**) Further analysis with simple linear regression indicated that there was no correlation between changes in basal calcium levels and changes in activity-induced calcium influx observed in response to acute hyperosmotic challenge, in either the proximal or distal dendrites of OT-MCNs (Pearsons’s r=-0.07, 0.06, p = 0.72, 0.72, respectively, regression lines in red). Similarly, the coefficient of determination (r^2^) indicated that changes in basal calcium was not predictive of changes in activity-induced calcium influx in either proximal or distal dendrites of OT-MCNs (r^2^=0.004, 0.003, respectively). Figure 4—figure supplement 2—source data 1.Excel file containing source data for panels A-C.

**Figure 4—figure supplement 3.**
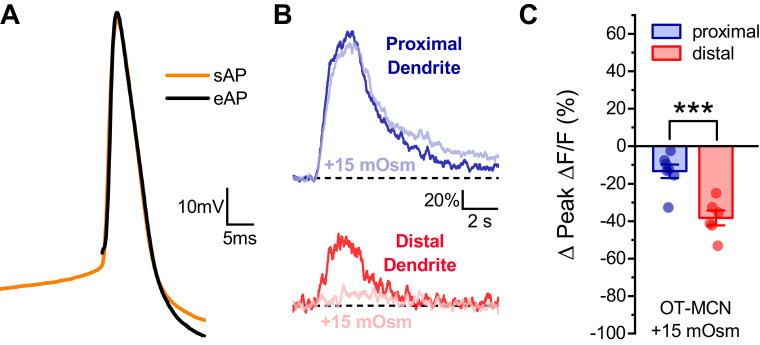
Effect of acute hyperosmotic challenge on activity-induced calcium influx in proximal vs. distal OT-MCN dendrites is preserved in neurons recorded in current clamp. (**A**) Raw voltage traces from a representative individual OT-MCN illustrating the similarity between a spontaneous action potential (sAP, orange trace) and an action potential evoked by a 250 μs suprathreshold current pulse (eAP, black trace). The current pulse used to evoke the eAP occurred immediately before the start of the illustrated trace, and was part of a train of identical pulses (delivered for 2 s at 20 Hz). Across all cells tested paired two-sample t-tests revealed no significant difference between spontaneous and evoked action potentials, respectively, in terms of peak voltage (38.0 ± 2.77 mV vs. 36.3 ± 1.39 mV, n=13, t = 0.66, p = 0.52), maximum rate of depolarization (102.14 ± 4.36 mV/ms vs. 112.6 ± 10.2 mV/ms, n=13, t = −0.83, p = 0.42), or maximum rate of repolarization (−31.8 ± 0.87 mV/ms vs. −32.2 ± 1.03 mV/ms, n=13, t = 0.53, p = 0.61). (**B**) Representative traces illustrating the effect of acute hyperosmotic challenge on activity-induced calcium influx, as observed in current clamp, in both proximal and distal dendrites (blue, red respectively). (**C**) Summary data indicates that hyperosmotic challenge had a small inhibitory effect on activity induced calcium influx as observed in the proximal dendrites of OT-MCNs (−13.3 ± 3.6% n = 7, t = −3.7, p = 0.01, one-sample t-test), while also producing a significantly larger inhibitory effect in the distal dendrites (−38.3 ± 3.92%, n=7, *** indicates t = 4.68, p = 0.0007, proximal effect vs. distal effect, unpaired two-sample t-test). These effects were not significantly different than observed in voltage clamped neurons in Figure 4 (proximal: VC, IC: −14.30 ± 3.37,–13.33 ± 3.61, n=13, 7, respectively, t = −0.18, p = 0.86, unpaired two-sample t-test, distal: VC, IC: −40.63 ± 4.23,–38.27 ± 3.92, n=20, six respectively, t = −0.29, p = 0.77, unpaired two-sample test). Figure 4—figure supplement 3—source data 1.Excel file containing source data for panel C.

Finally, we tested the effect of removing external calcium on activity-induced calcium influx as observed in OT-MCN dendrites. Specifically, we found that a 15 min exposure to calcium-free ACSF (see Materials and methods) reduced activity-induced calcium influx by 80.3 ± 3.5% when measured in the proximal dendrite (~25 µm from the soma, n=5, t = −22.9, p = 2.15 x 10^−5^, one-sample t-test), and by 67.9 ± 3.3% when measured in the distal dendrite (~125 µm form the soma, n=6, t = −20.75, p = 4.81 x 10^−6^, one-sample t-test). Both effects rapidly recover when extracellular calcium is restored. These results, presented in Figure 3—figure supplement 1, indicate that external calcium influx is the primary driver of the calcium signal observed in OT-MCN dendrites after somatic stimulation.

### Acute hyperosmotic challenge preferentially reduces activity-induced calcium influx in distal vs. proximal dendrites of OT-MCNs

We next tested the hypothesis that acute hyperosmotic challenge (as produced by bath application of 15 mOsm MT) directly modulates the relationship between somatic activity and activity-induced dendritic calcium influx in OT-MCNs. Toward that end, we used a technical approach similar to that employed in Figure 3; however, instead of measuring activity-induced influx at multiple locations under control conditions, we picked just two dendritic locations, proximal and distal to the soma, and repeatedly measured activity-induced calcium influx before and after acute hyperosmotic challenge. Proximal dendritic locations were again defined as being ~25 µm from the soma, while distal ones were located at ~125 µm from the soma (See Figure 4A, blue and red dashed lines, respectively). In order to generate activity-induced calcium influx at these locations the soma was stimulated with the same 2 s 20 Hz train of action potential like voltage steps as used in Figure 3, 2P line scan data were collected from each dendritic location and analyzed in an identical manner, and experiments were again performed in the continuous presence of bath applied antagonists for glutamate and GABA receptors (see previous Results section and Materials and methods). We found that acute hyperosmotic challenge had a small inhibitory effect on activity-induced calcium influx in the proximal dendrites of OT-MCNs (−14.3 ± 3.37%, n = 13, t = −4.2, p = 0.001, one-sample t-test), but had a much larger inhibitory effect in the distal dendrites (−40.6 ± 4.23%, n = 20, t = 4.44, p = 1.1 x 10^−4^ vs. proximal, Figure 4B, Figure 4C, left panel, see Figure 4—figure supplement 1 for data separated by sex). Notably, these changes were not associated with any significant/detectable impact on somatic membrane resistance (baseline: 1355 ± 109.8 MΩ, after MT: 1256 ± 109.3 MΩ, n=38, t = 1.38, p = 0.18, paired two-sample t-test), or on the voltage clamp current applied to the soma during the train (94.9 ± 5.21% of baseline after MT, n=11, t = −1.0, p = 0.4, one-sample t-test). Considered together, these observations suggest a site of action along the dendritic membrane.

Although glutamate and GABA receptors were antagonized during the experiments described above, it was possible that increased osmolarity might promote action potential dependent release of other modulators which then act locally on OT-MCN dendrites to reduce activity-induced calcium influx. In order to test this possibility, we repeated the experiment above with 1 µM TTX in the bath (in addition to glutamate and GABA receptor antagonists). However, under these conditions, we found that acute hyperosmotic challenge continued to robustly and preferentially inhibit activity-induced calcium influx in the distal vs. proximal dendrites (by −55.8 ± 6.53 vs. −10.5 ± 11.0%, distal, proximal, n=9, nine respectively, t = 4.51, p = 0.002, Figure 4C, right panel), suggesting activity-induced release of other modulators is not required. Data from this same experiment further revealed that the ratio of distal to proximal calcium influx within individual OT-MCNs in response to somatic stimulation did not change with bath application of TTX (n=5, ratio = 0.52 ±. 1 and 0.40 ± 0.1 before and after bath application of TTX respectively, t = 1.43, p = 0.23, paired two-sample t-test). This result further indicates that TTX-sensitive voltage-gated sodium channels play no apparent role in supporting propagation of voltage in OT-MCN dendrites.

Next, in order to eliminate any unexpected impact of the extra time required to change bath conditions in this experiment, we measured activity-induced calcium influx exclusively in the distal dendrites after 15 and 25 min of recording under constant bath conditions and noted that there was no significant change when measured at either time point (−2.0 ± 3.8%, 4.6 ± 5.3%, at 15 and 25 min, respectively, n=4, 4; t = −0.52, 0.86; p = 0.64, 0.45, one-sample t-tests, Figure 4D), or when measured across time points (n=4, 4, t = −1.28, p = 0.29, paired two-sample t-test). Similarly, we asked whether acute hyperosmotic challenge caused any change in basal calcium levels prior to somatic stimulation, and whether any such changes would impact subsequent measurement of activity-induced calcium influx. We found that on average, basal calcium levels changed by ≤ 6.1% in response to acute hyperosmotic challenge, and that these small changes were not correlated with observed effects on activity-induced calcium influx (Figure 4—figure supplement 2). Finally, we also evaluated the effect of acute hyperosmotic challenge on activity-induced calcium influx in OT-MCNs in current clamp, using a train of suprathreshold 250 μsec current pulses, delivered to the soma at 20 Hz for 2 s. We noted that within each individual OT-MCN, action potential shape and amplitude observed during this stimulus closely matched that observed during spontaneous firing (e.g. see Figure 4—figure supplement 3A). Further, the effects of acute hyperosmotic challenge on activity-induced calcium influx as observed in both proximal and distal dendrites were comparable to those observed in voltage clamp (proximal: −13.3 ± 3.61%, distal: −38.3 ± 3.92%, n=7, 6, t = 4.68, p = 0.0007, Figure 4—figure supplement 3B–C). Collectively, these data indicate that despite having an excitatory effect on action potential frequency as observed in the soma (Figure 2), acute hyperosmotic challenge also preferentially reduces activity-induced calcium influx as observed in the distal vs. proximal dendrites of OT-MCNs.

### Effects of acute hyperosmotic challenge on activity-induced calcium influx in distal dendrites are likely mediated by changes in dendritic membrane resistance

**Figure 5.**
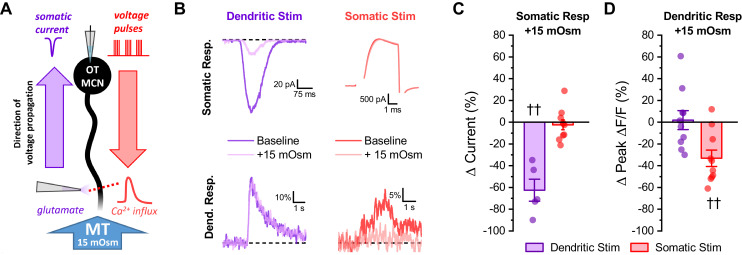
Acute hyperosmotic challenge selectively inhibits responses measured distal from a stimulus. (**A**) Diagram of experimental design. Two distinct stimuli (one to the distal dendrites, and one to the soma) were delivered every 15 s to an OT-MCN voltage clamped at −70 mV. The stimulus to the distal dendrites was local application of glutamate (bottom left), while the somatic stimulus was the same 2 s, 20 Hz train of action potential-like voltage pulses used in earlier experiments (delivered though the patch pipette, top right). For each stimulus, we used electrophysiological techniques (whole-cell recording) to measure the somatic response, and optical techniques (two-photon line scans across the distal dendrite as in earlier figures) to measure the dendritic response. Both the electrophysiological and optical responses to each stimulus were measured both before and after bath application of 15 mOsm MT. (**B**) Illustrates representative recordings, both before and after acute hyperosmotic challenge, for each combination of stimulus and response described. (**C**) Illustrates the effect of acute hyperosmotic challenge on somatic responses to either dendritic or somatic stimulation (purple, red, respectively). (**D**) Illustrates the effect of acute hyperosmotic challenge on dendritic responses to either dendritic or somatic stimulation (purple, red, respectively). Collectively these results highlight that bath application of 15 mOsm MT effectively and selectively inhibits responses that are measured distal from the stimulus that produced them, irrespective of whether those responses are measured with electrical or optical techniques. These data reinforce the conclusion that acute hyperosmotic challenge reduces dendritic membrane resistance. †† p<0.01, one-sample t-test. Figure 5—source data 1.Excel file containing source data for panels C-D.

To further test the hypothesis that acute hyperosmotic challenge decreases membrane resistance, and thus conductivity, in OT-MCN dendrites, we performed an additional control experiment. Specifically, we initiated whole-cell patch clamp recordings from OT-MCNs using the same internal and external solutions as was used above, except now without glutamate receptor antagonists in the bath. We then stimulated them alternately (every 15 s) with two distinct stimuli (one delivered to the soma and one to the distal dendrites). The somatic stimulus was the same 2 s 20 Hz train of action potential like voltage steps used in earlier experiments, while the dendritic stimulus was focal application of exogenous glutamate (accomplished using a picospritzer, see Materials and methods). For each stimulus, responses were measured in both the soma (as a whole-cell current) and in a distal dendrite (as ΔF/F generated with a 2P line scan). Collectively, this experimental design (Figure 5A-B) provides insight, in each individual OT-MCN tested, on how acute hyperosmotic challenge effects current propagating along the dendrite in both directions, either from the soma toward the distal dendrites, or from the distal dendrites toward the soma. Acute hyperosmotic challenge reduced somatic current observed in response to exogenous glutamate delivered to the distal dendrite by 62.5 ± 10.1% (n = 5, t = −6.17, p = 0.003, Figure 5C), but had no effect on somatic current observed in response to a train of AP like voltage pulses delivered to the soma (Δ current = −2.4 ± 4.53%, n = 10, t = −0.5, p = 0.612, Figure 5C). Conversely, acute hyperosmotic challenge reduced dendritic calcium influx produced by delivering a train of voltage pulses to the soma (by −33.13 ± 7.5%, n = 10, t = −4.4, p = 0.002, Figure 5D), but had no effect on dendritic calcium influx observed in response to locally applied glutamate (Δ Peak Δ F/F: 1.94 ± 8.7% of baseline, n = 10, t = 0.22, p = 0.828, Figure 5D). These results demonstrate that acute hyperosmotic challenge consistently and selectively inhibits whichever response is measured distal from the stimulus that produced it, irrespective of whether that response is measured using electrical or optical techniques. These results, in combination with other data presented above, effectively rule out the idea that the observed effects of acute hyperosmotic challenge depend on direct modulation of voltage-gated calcium channels, calcium induced calcium release, or on other aspects of calcium homeostasis in the dendrites. As such, they are consistent with the hypothesis that acute hyperosmotic challenge is directly modulating dendritic membrane resistance, and thus voltage propagation, in OT-MCNs. Finally, we attempted to directly measure changes in dendritic membrane resistance during an acute hyperosmotic challenge using dual patch clamp recordings from the soma and distal dendrites of a single OT-MCN. This approach has been used successfully to study the physiology of large dendrites in rat cortical pyramidal neurons and in cerebellar Purkinje cells; however, existing literature highlights that dendritic patching in smaller multipolar neurons is only feasible at proximal locations (Davie et al., 2006). Indeed, we found that the very small diameter and variable orientation of mouse OT-MCN distal dendrites, combined with tissue movement associated with changes in osmotic pressure, prohibited direct measurement of dendritic membrane resistance in this manner. Thus, we conclude that the combined electrical / optical approaches used in this study effectively and reliably reveal novel aspects of dendritic physiology in OT-MCNs that are not readily accessible to direct electrical recording.

### Changes in dendritic membrane resistance produced by acute osmotic challenge are cell type specific, compartment specific, and bidirectional

**Figure 6.**
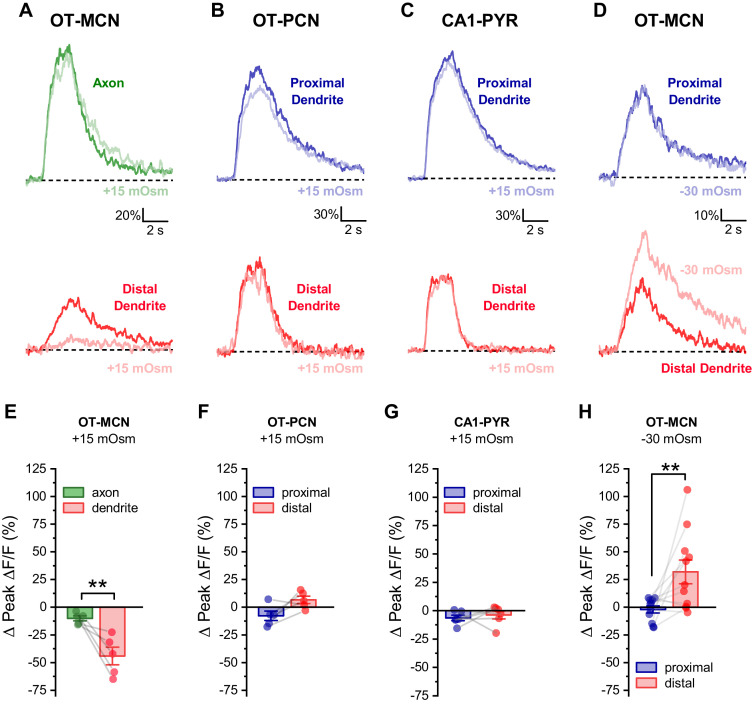
Effect of acute hyperosmotic challenge is compartment-specific, cell type-specific and bidirectional. (**A**) Compartment specificity: Calcium responses as observed in a representative cell indicating that acute hyperosmotic challenge preferentially inhibits activity-induced calcium influx in the distal dendrites as opposed to the distal axon of OT-MCNs. (**B–C**) Cell type specificity: Representative traces indicating that, in contrast to results reported for OT-MCNs (Figure 4), acute hyperosmotic challenge does not preferentially or selectively inhibit calcium influx (observed using identical techniques) in the distal dendrites of either PVN OT-PCNs (panel **B**) or hippocampal CA1 pyramidal neurons (panel **C**). (**D**) Bidirectionality: Representative traces indicating that, in contrast to results reported in Figure 4, acute hypo- (rather than hyper-) osmotic challenge selectively and preferentially increases (rather than decreases) activity-induced calcium influx observed in the distal dendrites of OT-MCNs. (Panels **E-H**) illustrate summary data for experiments represented in panels A-D, respectively. ** indicates p≤0.01, paired two-sample t-test. Figure 6—source data 1.Excel file containing source data for panels E-H.

Next, to eliminate any possible generalized or nonspecific effects of acute hyperosmotic challenge, we designed experiments to test the hypothesis that the effects are both compartment specific and cell type specific. Compartment specificity was evaluated using techniques identical to those employed for Figure 4, except that we compared activity-induced calcium influx in the distal dendrite to that observed in the distal axon. We found that acute hyperosmotic challenge again effectively inhibited activity-induced calcium influx in distal dendrites (Δ Peak Δ F/F: −44.0 ± 8.0%, n = 5, t = −5.5, p = 0.005), but produced a much smaller effect in the distal axon (Δ Peak Δ F/F: −10.0 ± 2.3%, n = 5, t = 3.4, p = 0.027 vs. distal dendrite, Figure 6A,E). This result demonstrates significant compartment specificity within individual OT-MCNs. In order to evaluate cell type specificity, we used identical approaches to measure activity-induced calcium influx in proximal vs. distal dendrites in PVN OT-PCNs (identified as described in Figure 1), and in CA1 pyramidal cells. In OT-PCNs, there was no significant effect of acute hyperosmotic challenge in proximal or distal dendrites (Δ Peak Δ F/F: −7.7 ± 4.3%, n = 5, t = −1.8, p = 0.147; 6.6 ± 3.4%, n = 5, t = 1.96, p = 0.12, respectively, Figure 6B,F). In CA1 pyramidal cells, we noted a weak inhibitory effect in proximal dendrites, but no effect in distal dendrites (proximal: −6.4 ± 2.4%, n=6, t = −2.7, p = 0.045; distal: −3.80 ± 3.5%, n = 6, t = −1.1, p = 0.33, Figure 6C,G). These results effectively demonstrate cell type specificity. Next, we reasoned that if acute hyperosmotic challenge inhibits calcium influx in the distal dendrites of OT-MCNs by opening a distinct dendritic osmosensitive ion channel, and if that channel is not completely closed in control conditions, then an acute hypoosmotic stimulus should have opposite effects. Indeed, we found that acute reduction of osmolarity by 30 mOsm (achieved by diluting the bath solution with water) effectively increased activity-induced calcium influx in the distal dendrites of OT-MCNs (by 32 ± 10.7% of baseline, n = 10, t = 3.0, p = 0.014, one-sample t-test), while having no effect in the proximal dendrites (−2.03 ± 3.18% of baseline, n = 10, t = −0.6, p = 0.536, one-sample t-test, Figure 6D,H). As with effects of acute hyperosmotic challenge, these changes occurred absent any significant effect on somatic membrane resistance or voltage clamp current observed during stimulation (n=10, t = 1.3, p = 0.21; n=10, t = −1.84, p = 0.1, respectively, one-sample t-tests). Note that the hypoosmotic stimulus used here will dilute extracellular calcium by ~10% (from ~2.4 mM to ~2.16 mM), which is expected to decrease driving force on calcium by < 2 mV. Collectively, these results demonstrate that the effects of acute osmotic challenge on activity-induced calcium influx, as observed in the distal dendrites of OT-MCNs, are compartment specific, cell type specific, and bidirectional.

### Changes in dendritic membrane resistance as produced by acute hyperosmotic challenge preferentially inhibit minimally evoked EPSCs produced by a distal vs. proximal stimulator

**Figure 7.**
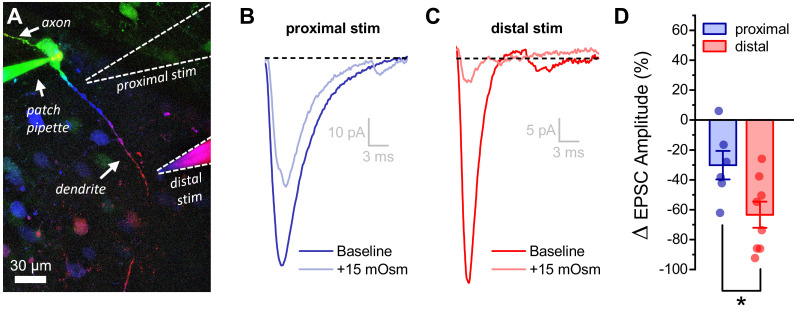
Acute hyperosmotic challenge has a greater inhibitory effect on distally vs. proximally evoked EPSCs in OT-MCNs. (**A**) Representative OT-MCN (depth-coded two-photon Z-series projection) illustrates placement of minimal stimulators used to evoke EPSCs at either proximal or distal dendritic locations. (**B–C**) Representative data illustrating evoked EPSCs produced by proximal (panel B) vs. distal stimulation (panel C), both before and after bath application of 15 mOsm MT. (**D**) Summary data indicates that on average, acute hyperosmotic challenge produces greater inhibition of distally evoked EPSCs. * p = 0.03 (n=6,8, proximal, distal, respectively, unpaired two-sample t-test). Figure 7—source data 1.Excel file containing source data for panel D.

The observation that the somatic response to activation of glutamate receptors on the distal dendrites of OT-MCNs is significantly inhibited by acute hyperosmotic challenge, in combination with other results above, suggests that changes in dendritic membrane resistance are likely to impact integration of synaptic inputs, as well as activity-induced calcium influx. In order to test this hypothesis directly we used minimal stimulation techniques (see Materials and methods) to evoke glutamate release from one or few axons that make synaptic contact with either the proximal or distal dendrites of an OT-MCN (Figure 7A). After identifying a clear evoked excitatory postsynaptic current (eEPSC), we bath applied 15 mM MT as in prior experiments. We found that acute hyperosmotic challenge reliably and reversibly reduced eEPSC amplitude as evoked by a minimal stimulator placed near the distal dendrite (by 63.4 ± 8.7%, n = 8, t = −7.3, p = 1.6 x 10^−4^, one-sample t-test Figure 7A,C). As in earlier experiments, this result was not associated with a significant change in somatic membrane resistance (n=8, t = −0.14, p = 0.90, paired two-sample t-test). If, as expected, the effect is instead produced primarily by a drop in dendritic membrane resistance, then the same acute osmotic stimulus should have less of an inhibitory effect on EPSCs generated using a minimal stimulator placed near the proximal dendrite. Indeed, consistent with this hypothesis, we found that 15 mM MT reduced proximally evoked EPSC amplitude by 30 ± 9.6% (n=6, t = −3.16, p = 0.03, Figure 7A,B). Consistent with our hypothesis, this effect is significantly smaller than observed when using a stimulator placed near the distal dendrite (Figure 7D, n=6, 8, proximal, distal, respectively, t = 2.55, p = 0.03). In order to confirm that eEPSCs involved in these experiments were glutamatergic, a subset of MT-sensitive responses (n=3) were challenged with bath applied glutamate receptor antagonists after recovery, and were effectively eliminated (not illustrated).

### Distinct somatic vs. dendritic effects of acute hyperosmotic challenge on OT-MCNs are likely to depend on compartment specific and molecularly distinct osmosensors

**Figure 8.**
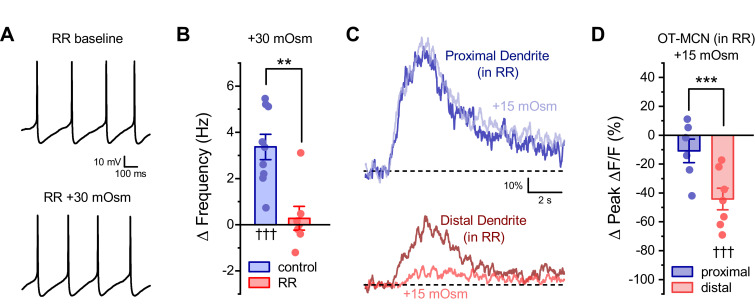
Ruthenium red selectivity blocks the somatic, but not dendritic, effect of acute hyperosmotic challenge. (**A**) Raw data from a representative OT-MCN illustrating spontaneous firing before and after acute hyperosmotic challenge in the continuous presence of TRPV antagonist ruthenium red (RR). (**B**) Summary data indicating that the effect of acute hyperosmotic challenge on action potential firing frequency in OT-MCNs (blue bar, ††† indicates p <0.001, one-sample t-test) is blocked in cells pretreated with RR (red bar, *** indicates p = 0.001, two-sample unpaired t-test, see text for further details). (**C**) Representative traces indicating that acute hyperosmotic challenge preferentially reduced activity-induced calcium influx in the distal vs. proximal dendrites of OT-MCNs. (**D**) In slices pre-treated with RR, acute hyperosmotic challenge had no significant effect on activity-induced calcium influx as observed in the proximal dendrites (ΔPeak ΔF/F: −10.9 ± 8.12%, n=6, t = −1.34, p = 0.24, one-sample t-test), but significantly reduced it in the distal dendrites (ΔPeak ΔF/F: −44.24 ± 7.47%, n=7, t = −5.92, p = 0.001, one-sample t-test, indicated by †††). *** indicates t = 3.03, p = 0.012 for effect in distal vs. proximal dendrites, unpaired two-sample t-test. Effects observed in this experiment also did not differ when compared to effects observed absent RR and reported in Figure 4C (proximal vs. proximal in RR: t = −0.37, p = 0.72, distal vs. distal in RR: t = 0.43, p = 0.67, unpaired two-sample t-test in both cases). Note that the control dataset in panel 8B above was previously presented in Figure 2C. ** indicates p = 0.001, unpaired two-sample t-test. Figure 8—source data 1.Excel file containing source data for panels B.

The observation that acute hyperosmotic challenge preferentially inhibits activity-induced calcium influx in the distal vs. proximal dendrites of OT-MCNs suggests that there are functional osmosensitive ion channels expressed along the length of the dendritic membrane. By contrast, the observation that the same osmostic stimulus produces an increase in spontaneous firing frequency of OT-MCNs as measured in current clamp, suggests that functional somatic osmosensors also exist. Because the somatic effect on spontaneous firing frequency requires depolarization, but the dendritic effect on activity-induced calcium influx does not significantly alter basal calcium levels, we hypothesized that somatic and dendritic osmosensors are likely to be molecularly distinct. Significant prior work has indicated that vasopressinergic MCNs are osmosensitive and has further revealed that transient receptor potential subfamily V member 1 (TRPV1) channels contribute to their osmosensitivity (Sharif Naeini et al., 2006; Bourque, 2008; Moriya et al., 2015; Prager-Khoutorsky and Bourque, 2015). Therefore, we evaluated the effect of ruthenium red (RR), a generic antagonist of transient receptor potential (TRP) ion channels, on both somatic and dendritic effects of acute hyperosmotic challenge observed in OT-MCNs. We found that pre-treatment with 10 µM RR blocked the effect of acute hyperosmotic challenge on basal firing rate as observed in current clamp (Δ action potential frequency: 0.28 ± 0.21, n=7, t = 0.5, p = 0.61, t = 4.0, p = 0.001 vs. response to same stimulus absent RR, Figure 8A–B). Conversely, RR did not alter the effect of acute hyperosmotic challenge on activity-induced calcium influx as observed in either the proximal or distal dendrites (Figure 8C–D, see text of legend for further details). Similarly, RR also did not block the inhibitory effect of acute hyperosmotic challenge observed on distally evoked EPSCs (Δ EPSC amplitude: −71.5 ± 6.0%, n=6, t = −11.9, p<0.001, t = 0.72, p = 0.49 vs. effect observed absent RR, See Figure 8—figure supplement 1). Collectively, these results reinforce the hypothesis that PVN OT-MCNs express distinct osmosensors in somatic vs. dendritic compartments and suggest that the somatic but not dendritic osmosensor may be a member of the TRPV family (see Discussion). Interestingly, we also noted that the effect of MT on activity-induced calcium influx as observed in the distal dendrites of OT-MCNs is blocked in cells filled with a cesium-gluconate internal solution, which suggests that the dendritic osmosensor may be cesium sensitive (See Figure 8—figure supplement 2 for additional details).

**Figure 8—figure supplement 1.**
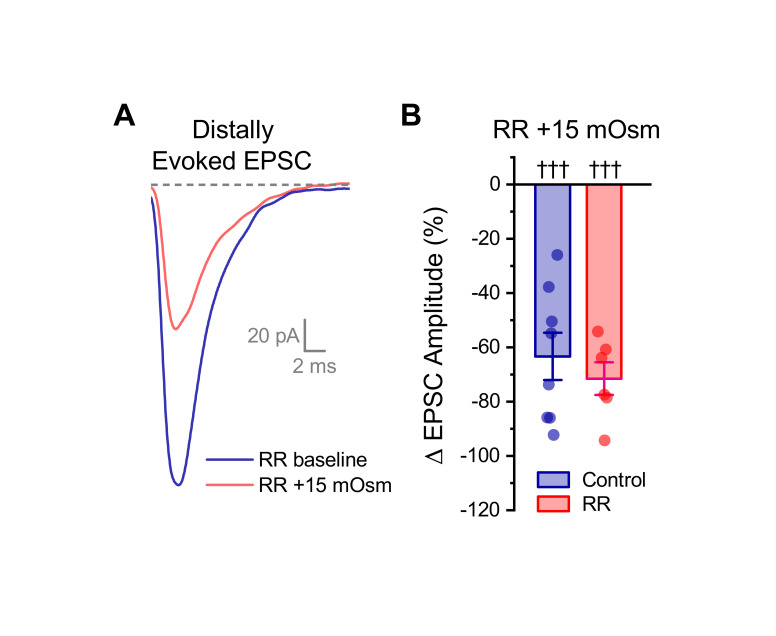
Ruthenium red does not block the effect of acute hyperosmotic challenge on distally evoked EPSCs. (**A**) Raw data from a representative OT-MCN illustrating that acute hyperosmotic challenge continues to inhibit evoked EPSCs generated with minimal stimulation near the distal dendrite, even in cells pretreated with RR. (**B**) Summary data highlighting that pretreatment with RR has no impact on the ability of acute hyperosmotic challenge to inhibit distally evoked EPSCs. Note that the control dataset in panel B was previously presented in Figure 7D. ††† indicates p≤0.001, one-sample t-test. Figure 8—figure supplement 1—source data 1.Excel file containing source data for panel B and D.

**Figure 8—figure supplement 2.**
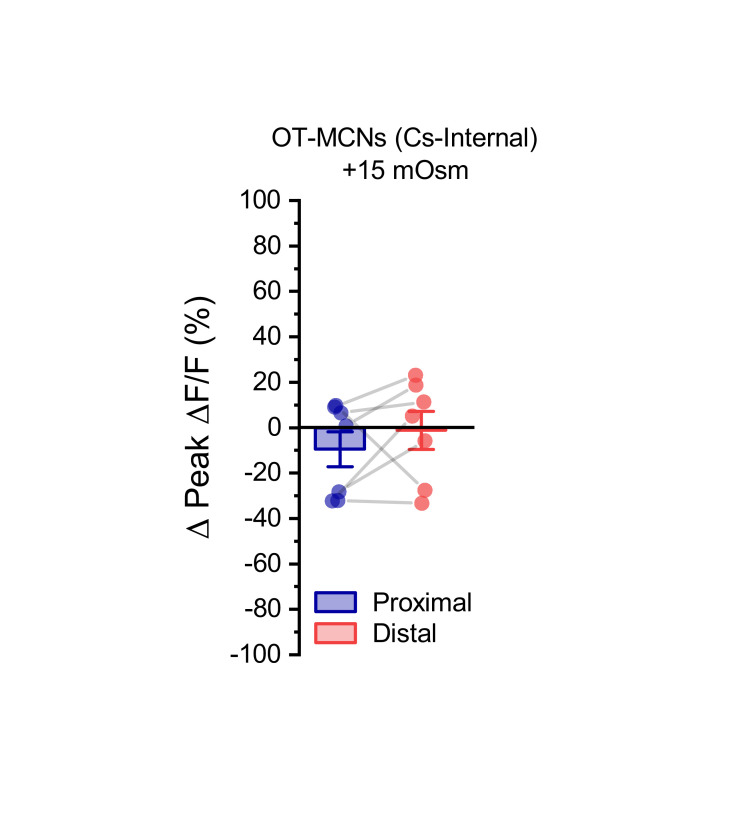
Acute hyperosmotic challenge has no effect on activity-induced calcium influx observed in dendrites of OT-MCNs filled with a Cs-gluconate-based internal solution. We noted that when the experiment presented in Figure 4A–C is conducted in OT-MCNs filled with a Cs-gluconate rather than a K-gluconate-based internal solution, acute hyperosmotic challenge no longer had any significant effect on activity-induced calcium influx as observed in either the proximal or distal dendrites (Proximal:. −9.5 ± 7.7%, Distal: −1.2 ± 8.3%, n=seven is both cases, t = −1.2,–0.1, p = 0.26, 0.9, respectively, using one-sample t-test, and group means were also not significantly different, t = −0.93, p = 0.39, paired two-sample t-test). Figure 8—figure supplement 2—source data 1.Excel file containing source data.

### GABA_A_ receptor activation preferentially reduces activity-induced calcium influx in distal vs. proximal dendrites of OT-MCNs

**Figure 9.**
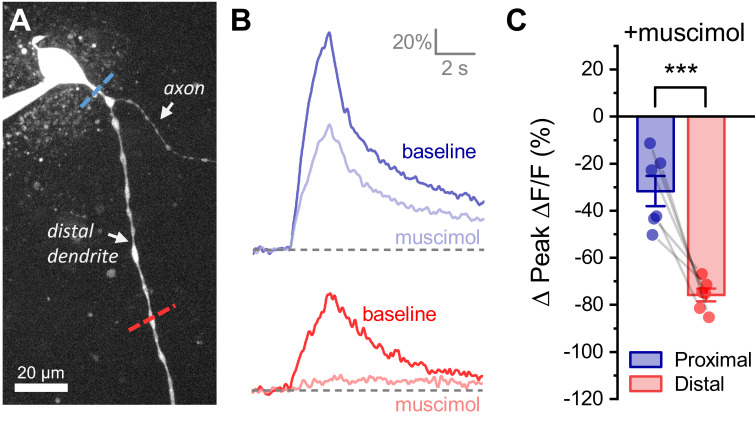
Activation of GABA_A_ receptors preferentially inhibits activity-induced calcium influx in distal vs. proximal dendrites of OT-MCNs. (**A**) Calcium responses to somatic activity were measured at proximal and distal sites (dashed lines) before and after bath application of 300 nM muscimol (a GABA_A_ receptor agonist). Other than using muscimol in place of acute hyperosmotic challenge, techniques are identical those described for Figure 4. (**B**) Representative calcium responses observed during somatic stimulation in the proximal (blue) vs. distal (red) dendrite of an OT-MCN, before (lighter trace) and after (darker trace) bath application of muscimol. (**C**) Summary data indicates that bath application of muscimol produces greater inhibition of activity-induced calcium influx in the distal vs. proximal dendrites of OT-MCNs. *** p<0.001, paired two-sample t-test. Figure 9—source data 1.Excel file containing source data for panel C.

Next, we sought to determine whether dendritic membrane resistance in OT-MCNs is an aspect of dendritic physiology that can be actively manipulated to modify the relationship between somatic activity and dendritic calcium influx, even under conditions that do not involve changes in osmolarity. Toward that end, we performed experiments similar to those presented in Figure 4, except we replaced the hyperosmotic stimulus with bath application of 400 nM muscimol (a GABA_A_ receptor agonist). Unlike acute hyperosmotic challenge, bath application of muscimol significantly reduced somatic membrane resistance (from 1178 ± 166.6 MΩ to 226 ± 43.5 MΩ, n = 6, t = 4.9, p = 0.004, paired two-sample t-test) and produced a tonic inhibitory current (of 15.4 ± 5.42 pA, n = 6, t = 2.8, p = 0.036, one-sample t-test) apparent in cells voltage clamped at −70 mV, consistent with tonic activation of somatic GABA_A_ receptors. However, importantly, like acute hyperosmotic challenge, muscimol preferentially inhibited activity-induced calcium influx in the distal vs. proximal dendrites (−75.8 ± 2.7% vs. −31.6 ± 6.4%, respectively, n=6, t = 5.6, p = 0.003, Figure 9). This finding is consistent with opening of GABA receptors along the dendritic membrane (see also Pirker et al., 2000; Park et al., 2006), and notably, highlights that dendritic membrane resistance is likely to be under constant and dynamic regulation in OT-MCNs even absent changes in osmolarity.

## Discussion

This study uses a combination of electrical and optical recording techniques to examine activity-induced calcium influx in the dendrites of PVN OT-MCNs in OT-tdTomato reporter mice. Somatic activity was induced with a well-controlled train of action potential like stimuli delivered to the soma, while activity-induced calcium influx was measured in OT-MCN dendrites using quantitative two-photon microscopy. We demonstrate that PVN OT-MCN dendrites are weak conductors of somatic voltage changes in basal conditions in both male and virgin female mice, and importantly, we also find that activity-induced calcium influx in the dendrites is subject to robust modulation by a diverse set of stimuli acting on distinct types of ion channels in the dendritic membrane.

The primary stimulus used in the current study is an acute increase in osmotic pressure. Hyperosmotic stimuli are well-recognized for an ability to increase activity of hypothalamic MCNs, and in so doing, for promoting significant increases in both peripheral and central OT concentration (Mason, 1980; Bourque and Renaud, 1984; Ludwig et al., 1994; Ludwig, 1998; Tasker et al., 2020). Increases in peripheral OT concentration are understood to depend on axonal release in the neurohypophysis (which delivers OT directly into the vasculature), while increases in central concentration are likely to depend heavily on dendritic release into the extracellular space, the subarachnoid space, and/or the third ventricle (Bourque, 1991; Ludwig and Leng, 2006; Veening et al., 2010). A curious feature of the neurohormonal response to hyperosmotic challenge is that increases in peripheral OT concentration are both rapid and frequency dependent (with concentration closely tracking MCN activity), while increases in central OT concentration are temporally separated from peak MCN firing, often by over an hour (Ludwig et al., 1994; Ludwig, 1998). This is somewhat counter intuitive because like axon terminals, OT-MCN dendrites also contain many large dense core vesicles loaded with oxytocin, and these dendritic vesicles are also subject to both activity and calcium-dependent release (Mason et al., 1986; Pow and Morris, 1989; Ludwig et al., 1995; Wang et al., 1995). Notably, other types of peripheral stimuli (e.g. suckling) can drive more concurrent increases in peripheral and central OT concentration suggesting more synchronous release of OT from both axon terminals and dendrites (Neumann et al., 1993), while some locally synthesized signaling molecules (e.g. α-melanocyte stimulating hormone) have been demonstrated to promote dendritic release of OT while actively inhibiting both firing rate and release from axon terminals in the neurohypophysis (Neumann et al., 1993; Sabatier et al., 2003; Sabatier, 2006). Collectively, these types of data effectively highlight that although at least loosely coupled, the relationship between somatic activity and dendritic release of peptide in hypothalamic MCNs is likely subject to modulation.

The most well-established basis for understanding this flexibility invokes a model of conditional priming, whereby specific endogenous modulators, acting directly on the dendrites, can prime dendritic vesicles to be more available for activity-induced release (Morris and Ludwig, 2004; Ludwig and Leng, 2006). However, in the specific case of hyperosmotic challenge, maximizing calcium-dependent dendritic priming prior to hyperosmotic challenge was found to increase the amount of dendritic release of OT, remarkably, without altering its time course (Ludwig et al., 2002). This striking result suggests that there must be some aspect of OT-MCN physiology that is activated by hyperosmotic challenge, that is separate from conditional priming, and that is capable of rapidly yet transiently reducing the probability of activity-induced exocytosis of dendritic OT.

In that regard, a key finding of the current study is that an acute hyperosmotic stimulus delivered in vitro decreases activity-induced calcium influx in OT-MCN dendrites, while an acute hypoosmotic stimulus increases it. In each case, we noted minimal effect of changing bath conditions on somatic input resistance, or on somatic current observed during the stimulus, and further noted that changes in osmolarity had a more robust effect in distal vs. proximal dendrites. We noted no similar inhibitory effect of acute hyperosmotic stimuli on activity-induced calcium influx in the distal axons of OT-MCNs, indicating compartment specificity, or in the distal dendrites of OT-PCNs or CA1 pyramidal cells, demonstrating cell-type specificity. Further, in experiments that involved both somatic and dendritic stimulation techniques, we demonstrated that hyperosmotic challenge selectively inhibits responses measured distal from the stimulus, irrespective of whether those measurements are made using electrical or optical techniques. Collectively, these data indicate for the first time that osmolarity modulates activity-induced calcium influx in OT-MCN dendrites by acting on osmosensitive ion channels expressed along the dendritic membrane. Based on these data, it is reasonable to postulate that this mechanism may reduce activity-induced dendritic release of OT during times of high somatic activity as induced by acute hyperosmotic challenge. As such, it may be interesting for future studies to evaluate whether low levels of dendritic calcium influx produced by somatic activity when dendritic osmosensitive channels are open, or concurrent action of other dendritic modulators, helps promote priming of dendritic vesicles in a way that contributes to enhanced dendritic release once basal osmolarity is restored.

Another aspect of this study worth specifically highlighting is the novel implication that OT-MCNs express molecularly distinct osmosensitive channels in their soma vs. dendrites. This conclusion is supported by the observation that a non-selective TRP ion channel receptor antagonist, RR, blocked the effect of acute hyperosmotic challenge on action potential firing frequency without altering effects on activity-induced calcium influx as observed in either the proximal or distal dendrites, or the effects on evoked EPSCs produced by a minimal stimulator placed near the distal dendrite. RR is an antagonist of TRPM6, TRPA1, TRPC3, and of all members of the TRPV family (TRPV1-6, Clapham, 2007; Lichtenegger and Groschner, 2014). That said, to our knowledge TRPV channels are the only RR sensitive channels that have potential for osmosensitivity and that are strongly expressed in the hypothalamus (Sharif Naeini et al., 2006; Sladek and Johnson, 2013; Prager-Khoutorsky and Bourque, 2015; Zaelzer et al., 2015; Shenton and Pyner, 2018), suggesting that the somatic osmosensor described here may be a member of the TRPV family. Additional potential effects of RR, for example on ryanodine receptors or mitochondrial calcium transporters, are not expected to be a factor in current experiments since RR is membrane impermeant (Luft, 1971a; Luft, 1971b; Garcha and Hughes, 1994), and we only applied it extracellularly. Additional evidence for the hypothesis that OT-MCNs have distinct somatic and dendritic osmosensors comes from the observation that intracellular cesium blocked the effects of acute hyperosmotic challenge on activity-induced calcium influx in the distal dendrites even through TRPV receptors are cesium permeant (Caterina et al., 1997; Puopolo et al., 2013). Thus, overall, we hypothesize that the somatic osmosensor described here is a member of the TRPV family, while the dendritic osmosensor is, or is coupled to, a cesium sensitive and potassium permeant channel. While some prior literature is generally consistent with these hypotheses (Liu et al., 2005; Sharif Naeini et al., 2006; Zhang et al., 2009; Prager-Khoutorsky and Bourque, 2015), very few studies have examined these questions with respect to OT-MCNs in particular.

Next, it is interesting to highlight that all novel aspects of OT-MCN dendritic physiology revealed here, as well as most other core intrinsic features of OT-MCNs observed, were identical in male and virgin female mice, suggesting that they have a fundamental and sex independent role in regulation of oxytocinergic signaling. That said, it seems plausible that aspects of OT-MCN physiology likely relevant to central OT signaling, such as the dendritic conductivity, could be regulated in a context and sex specific way. As such, it may be interesting to determine whether activity-induced calcium influx in the distal dendrites of OT-MCNs is altered in pregnant or lactating females. Indeed, concurrent increases in both peripheral and central release of OT have been reported in response to suckling (Neumann et al., 1993).

Other aspects of this study make two additional important points. First, we demonstrate directly that the effects of acute hyperosmotic challenge on OT-MCN dendrites are not limited to modulation of activity-induced calcium influx, but also robustly inhibit the somatic response to endogenous synaptic inputs arriving at the distal dendrites. This finding, in combination with other observed effects, suggests that activation of dendritic osmosensors not only transiently reduces the probability of activity-induced dendritic release of OT into the CNS, but also simultaneously reduces the impact of descending central inputs forming dendritic synapses on MCN firing rate. These changes are expected to effectively but transiently prioritize activity-induced increases in peripheral OT concentration. Second, we report that an ability to modulate activity-induced calcium influx by acting on dendritic ion channels can also be produced by stimuli that do not alter osmolarity. Specifically, we note that bath application of a GABA_A_ receptor agonist not only decreases somatic membrane resistance in OT-MCNs but also preferentially inhibits activity-induced calcium influx in the distal vs. proximal dendrites. The later finding is consistent with prior reports that multiple types of GABAergic receptor subunits, including the typically extrasynaptic δ subunit, are expressed on MCN dendrites and/or in magnocellular regions of the PVN (Fenelon et al., 1995; Pirker et al., 2000; Belelli et al., 2009), and is interesting to consider along with the fact tonic GABAergic currents have been directly observed in MCNs (Park et al., 2006). It is also important in the context of this study because it highlights that endogenous regulation of dendritic conductivity may represent an important mechanism for regulating central OT concentration even in situations where osmolarity remains constant. For example, it is intriguing to note that OT-MCNs receive significantly more frequent bursts of GABAergic IPSCs during lactation (Popescu et al., 2019), and that lactation has also been associated with a significant positive shift in the reversal potential of GABA-receptor-mediated currents (Lee et al., 2015). Overall, we expect that it will be important for future studies to evaluate the ability of additional endogenous modulators to impact conductivity of OT-MCN dendrites, and to identify specific contexts in which such modulators are active.

Finally, as noted in more detail in the Introduction, central OT signaling systems are a promising therapeutic target for a variety of conditions impacting mental health, and yet the best available current strategy for therapeutically modulating activation of central OTRs in humans involves intranasal delivery of an exogenous agonist that has low permeability to the blood brain barrier (Evans et al., 2014; Leng and Ludwig, 2016; Quintana and Woolley, 2016; Quintana et al., 2018). In our view, a better understanding of OT-MCN physiology, particularly as it relates to release of endogenous OT in the CNS, may ultimately lead to improved therapeutic options that are better able to mimic natural concentration, kinetic, and context-dependent aspects of central OT signaling. In the current study, measures of activity-induced calcium influx in OT-MCN dendrites reveals a novel aspect of dendritic physiology likely to underlie the flexible relationship between somatic activity and dendritic release of OT. Future studies may develop new methods for spatially precise quantification of dendritic exocytosis and/or for detection of quantal amounts of OT release very close to the dendritic membrane, which would in turn promote a more direct evaluation of the relationship between dendritic calcium influx and dendritic exocytosis.

## Materials and methods

**Key resources table:** 

Reagent type (species) or resource	Designation	Source or reference	Identifiers	Additional information
Strain, strain background (Mouse)	B6.129S-Oxt^tm1.1(cre)Dolsn^/J	Jackson Laboratory	JAX stock #024234; RRID:IMSR_JAX:024234	
Strain, strain background (Mouse)	B6.Cg-Gt(ROSA) 26Sor^tm14(CAG-tdTomato)Hze^/J	Jackson Laboratory	JAX stock #007914; RRID:IMSR_JAX:007914	
Antibody	Anti-neurophysin1 (NP1, mouse monoclonal)	Dr. H. Gainer, National Institute of Health	PS-38	1:400
Antibody	Anti-mouse Alexa Fluor 647 (donkey, polyclonal)	Jackson ImmunoResearch Labs	715-606-151 RRID:AB_2340866	1:500
Chemical compound, drug	DNQX	TOCRIS	3212	
Chemical compound, drug	AP5	TOCRIS	3693	
Chemical compound, drug	picrotoxin	Sigma-Aldrich	P1675	
Chemical compound, drug	CGP-55845	TOCRIS	1248	
Chemical compound, drug	mannitol	Sigma-Aldrich	M4125	
Chemical compound, drug	muscimol	TOCRIS	0289	
Chemical compound, drug	glutamate	Sigma-Aldrich	G2834	
Chemical compound, drug	ruthenium red	Sigma-Aldrich	R275-1	
Chemical compound, drug	Fluo-5F	ThermoFisher	F14221	
Chemical compound, drug	Alexa Fluor 594	ThermoFisher	A10438	
Software, algorithm	NIS-Elements AR 5.02	Nikon	RRID:SCR_014329	
Software, algorithm	FIJI	Schindelin et al., 2012	RRID:SCR_002285	https://fiji.sc
Software, algorithm	pClamp 10	Molecular Devices	RRID:SCR_011323	
Software, algorithm	OriginPro	OriginLab Corporation	RRID:SCR_014212	
Software, algorithm	Python 3.6		RRID:SCR_008394	https://www.python.org
Software, algorithm	Prairie View 5.4	Bruker Scientific	RRID:SCR_017142	

### Animals

All experiments in this study were performed using 1- to 3-month-old OT-reporter mice which expresses red fluorescent protein (tdTomato) in oxytocinergic neurons. These mice were generated by crossing OT-IRES-Cre knock-in mice which express Cre targeted to the *Oxt* locus (Jackson Labs Stock #024234) with Ai14 mice that express tdTomato following a loxP-flanked stop cassette in the *Rosa26* locus under control of the CAG promoter (Jackson Labs Stock #007914). This same strategy has been used in several previous studies to facilitate identification of oxytocinergic neurons (Clipperton-Allen et al., 2016; Xiao et al., 2017). Throughout the course of this study, experiments were performed on approximately equal numbers of male and virgin female mice. Results of all core experiments, including immunohistochemical evaluation of OT-reporter animals, analysis of intrinsic properties of OT-MCNs, and the evaluation of the effects of acute osmotic challenge on OT-MCNs, observed both somatically and in the distal dendrites, were carefully compared across sex. Because we noted no major sex-based differences between males and virgin females in any of these experiments, data in all primary figures are combined across sex. See main text of results section, Figure 1—figure supplement 1, and Figure 4—figure supplement 1 for further details. All animals were group-housed on a 12 hr light/dark cycle, and all animal procedures were approved by the University of Florida Institutional Animal Care and Use Committee (IACUC).

### Immunohistochemistry

We used immunohistochemical techniques to quantify co-expression of tdTomato and oxytocin-neurophysin 1 (NP1). Two male and two female mice were anesthetized with sodium pentobarbital (1.56 mg/g i.p.) and transcardially perfused with 0.15 M NaCl followed by 4% paraformaldehyde. Brains were extracted and post-fixed for 4 hr in 4% paraformaldehyde, then transferred to a sucrose solution (30% sucrose in PBS) and stored at 4°C for at least 24 hr. Brains were then sectioned at 30 microns and stored at −20°C in cryoprotective solution (1 L of 0.1 M PBS supplemented with 20 g PVP-40, 600 mL ethylene glycol, and 600 g sucrose). Unless otherwise noted, immunohistochemistry was conducted at room temperature using free-floating sections in 12-well plates (3 mL/well) on an orbital shaker. Following five rinses (5 min each) with 50 mM potassium PBS (KPBS), tissue was incubated in blocking solution (KPBS with 2% normal donkey serum and 0.2% Triton X-100) for 1 hr. Subsequently, sections were incubated in a primary antibody against NP1 overnight at 4°C (mouse monoclonal, PS-38, Dr. H. Gainer, National Institute of Health, 1:400)(Ben-Barak et al., 1985; de Kloet et al., 2016). Following five rinses (5 min each) in KPBS, sections were incubated in the secondary antibody (Alexa 647 Donkey anti-mouse, Jackson Immuno 715-605-150, 1:500) for 2 hr. Following five rinses (5 min each) with KPBS, slices were mounted on glass slides, air dried overnight, and coverslipped with polyvinyl alcohol mounting medium. Images were captured and analyzed using a Nikon C2+ scanning confocal microscope and NIS-Elements AR 5.02 software. Identical imaging parameters were used for the acquisition and analysis all images.

### Acute brain slice preparation

Mice received an IP injection of ketamine (0.1 mL of 100 mg/mL, in sterile physiological saline) and were euthanized using a small animal guillotine. Brains were extracted and coronal sections 200 µm thick were made using a Leica VT1000 S vibratome. During this procedure, slices were submerged in ice-cold, sucrose-laden, artificial cerebrospinal fluid (ACSF) containing (in mM): 87 NaCl, 2.5 KCl, 1.25 NaH_2_PO_4_, 7 MgCl_2_, 10 dextrose, 0.5 CaCl_2_, 75 sucrose, and 25 NaHCO_3_. After sectioning was complete, brain slices containing the PVN were transferred to an incubator filled with a low-calcium high-magnesium ACSF designed to improve slice viability during the incubation period. That ACSF contained (in mM): 124 NaCl, 2.5 KCl, 1.23 NaH_2_PO_4_, 10 dextrose, 1 CaCl_2_, 3 MgSO_4_, and 25 NaHCO_3_. Both solutions were continuously saturated with 95% O_2_/5% CO_2_ and had a pH of ~7.3. After 30 min of incubation at 37°C, slices were passively equilibrated to room temperature for an additional 30 min (minimum) prior to use.

### In vitro electrophysiology

In preparation for in vitro electrophysiological experiments, slices were transferred from the slice incubator to a low turbulence perfusion chamber (JG-23W/HP, Warner Instruments) where they were continuously perfused a rate of 2 mL/min with ACSF containing (in mM) 126 NaCl, 11 dextrose, 1.5 MgSO_4_, 3 KCl, 1.2 NaH_2_PO_4_, 2.4 CaCl_2_, and 25 NaHCO_3_. This solution was continuously oxygenated with 95% O_2_ and 5% CO_2_, had a pH of ~7.3, and was maintained at 28°C. Where noted, calcium-free ACSF was identical, except that 2.4 mM CaCl_2_ was replaced with 3.6 mM NaCl to maintain osmolarity. tdTomato positive PVN neurons were identified using an Olympus BX51WI stereomicroscope that supported both infrared differential interference contrast (IR-DIC) and conventional epifluorescence microscopy. Both IR-DIC and epifluorescence images were acquired through an Olympus 40X water immersion objective (LUMPFL40XWI/IR-2, Olympus) using 12-bit IR CCD camera (QICAM Fast 1394) controlled by Fiji software (Schindelin et al., 2012). An X-Cite Series 120Q (Lumen Dynamics) light source coupled with an XF406 filter set (Omega Optical) was used for conventional epifluorescence imaging. Patch pipettes were prepared using a Flaming/Brown pipette puller (Sutter Instruments, P-97). Borosilicate glass capillaries (1.5 mm/0.8 mm) were pulled to produce patch pipettes with an open tip resistance of 4–6 MΩ when filled with an intracellular solution that contained (in mM): 1 MgCl_2_, 1 EGTA, 10 HEPES, 125 K-gluconate, 10 phosphocreatine, 2 Na_2_-ATP, 0.25 Na-GTP, adjusted to pH 7.25 and 295 mOsm. This solution was used for all experiments that involved whole-cell recording but not simultaneous calcium imaging. For experiments that required concurrent calcium imaging (see next section), EGTA was omitted and replaced with 0.3 mM Fluo-5F (ThermoFisher F14221) and 0.03 mM Alexa 594 (ThermoFisher A10438). Where noted, K-gluconate in this solution was replaced with equimolar Cs-gluconate. Whole-cell recordings were made using a Multiclamp 700B amplifier, Digidata 1440A digitizer, and Clampex 10.7 software (Molecular Devices). All whole-cell data were sampled at 20 kHz and low-pass filtered at 2 kHz. When necessary, synaptic responses were generated using a small tipped bipolar stimulator pulled from theta glass (~1–1.5 µm inner diameter), connected to a constant current stimulus isolator (0.2 msec pulse duration). Simulator placement and stimulation intensity were adjusted until an evoked response ≥ 50 pA could be reliably generated. On-line analysis of whole-cell patch clamp data was performed with custom software using Python 3.6 and the pyABF module. Off-line analysis was performed using custom software written by CJF in OriginC (OriginLab Corporation, Northampton, MA). Access resistance, input resistance and whole-cell capacitance were continuously monitored in voltage clamp using data generated with brief (200 msec) hyperpolarizing steps from −70 mV to −100 mV. Cells were excluded from analysis if they did not survive until experiments were completed, or if they suffered a sudden change in patch quality as indicated by large or sudden changes in either holding current or access resistance during the course of recording. All experiments were performed in the continuous presence of bath applied antagonists for kainate/AMPA receptors, NMDA receptors, GABA_A_ receptors, and GABA_B_ receptors (20 µM DNQX, 40 µM AP5, 100 µM picrotoxin (PTX), and 10 µM CGP-55845 (CGP), respectively), except for those that explicitly involved measuring evoked excitatory synaptic currents (EPSCs), or effects of an exogenous glutamate or GABA receptor agonist. Some experiments transiently delivered tetrodotoxin (TTX, 1 µM), muscimol (400 nM), or ruthenium red (RR, 10 µM) using a syringe pump in-line with the bath perfusion system. In experiments that involved changing osmolarity of the bath, mannitol (MT, in ACSF) or purified deionized water were also delivered using a syringe pump. Local application of glutamate was achieved using a Parker Picospritzer III (model R374-01C, 5–10 ms pulse duration, 10–20 psi), connected to a glass pipette, identical to those used for whole-cell recording, loaded with 100 mM glutamate solubilized in ACSF. All drugs used during in vitro experiments were obtained from TOCRIS except PTX and glutamate which were obtained from Sigma-Aldrich. Although stock solutions for PTX and CGP were dissolved in DMSO, total bath concentration of DMSO never exceeded ~0.1% and remained stable throughout experiments.

### Subcellular calcium imaging

Subcellular two-photon calcium imaging in OT-MCN axons and dendrites was accomplished using an Ultima laser scanner (Bruker Scientific, Billerica, MA), powered by a Mira Ultrafast Ti:sapphire laser and a Verdi 5W pump (both from Coherent, Inc Santa Clara, CA). The emission wavelength of the Mira was set to 810 nm to simultaneously excite the calcium sensitive indicator, Fluo-5F, and the calcium insensitive indicator, Alexa Fluor 594 (see above for detailed composition of internal solution). All cells were permitted to rest for 15 min following establishment of whole-cell configuration to ensure robust diffusion of fluorophore into dendrites before proceeding with calcium imaging experiments. Laser power reaching the slice during two-photon imaging was adjusted (using a model 350–80 pockels cell, Conoptics, Danbury, DT) to a level where the basal (unstimulated) signal on the green (Fluo-5F) channel was visible, but this value was not precisely measured. A 40x water immersion objective (LUMPFL40XWI/IR-2, Olympus) was used for all imaging experiments. Somatic activity was induced in cells voltage-clamped at −70 mV using a train of brief action potential like voltage steps (to +50 mV for five msec, delivered at 20 Hz for 2 s). Activity-induced calcium influx was measured in dendrites or axons using two-photon line scans run across the structure of interest at a known distance from the soma. Line scans were acquired at 85 Hz, beginning 2 s before somatic stimulation and continuing for a total of 11.9 s. Where mentioned in the text, the voltage clamp current applied to the soma during the train was quantified over time by measuring the area under the curve of the last pulse. Emissions from the calcium sensitive indicator (Fluo-5F, green) and the calcium insensitive indicator (Alexa Fluor 594, red) were separated at 575 nm with a dichroic mirror, and simultaneously measured by two separate photomultiplier tubes (PMTs, Hamamatsu R3896 SEL). Raw data from the PMTs was used to calculate the fluorescence ratio of calcium-sensitive to calcium insensitive fluorescence (F/F) over time. This curve was then baseline subtracted using the mean F/F as observed in a 1.2 s baseline period that occurred immediately before the stimulus, smoothed using a 0.5 s Gaussian-weighted moving window function, and reported as ΔF/F. Peak ΔF/F is the peak of the ΔF/F curve as observed in response to the somatic stimulus, while Δ Peak ΔF/F is used to describe the change in peak ΔF/F resulting from a change in bath conditions, expressed as a percentage of the baseline response. Dendrites were distinguished from axons by their larger diameter and by their enhanced passive fill with somatically delivered Alexa Fluor 594. On-line acquisition of line scan data was performed using Prairie View 5.4 software (Bruker Scientific, Billerica, MA). Off-line analysis of line scan data was performed using custom software written in C# and OriginC by SWH and CJF.

### Statistical analysis

Within-cell changes in individual parameters produced by an experimental procedure were evaluated with a two-tailed 1-sample Student’s t-test (null hypothesis mean = 1 or mean = 0, for normalized vs. baseline subtracted data, respectively). When comparing a parameter of interest across two independent groups of cells, or across two different dendritic locations, a two-tailed two-sample Student’s t-test was used (null hypothesis: Group one mean = Group two mean). Welch’s correction was applied in cases where population variance was significantly different between samples. To determine whether there were sex-based differences in the intrinsic properties of OT-MCNs, or in the effect of acute hyperosmotic challenge as observed in proximal vs. distal OT-MCN dendrites, a two-way ANOVA was used with either sex and cell-type, or sex and dendritic location, as factors, respectively. For evaluating whether distance from the soma impacted activity-induced calcium influx within a single-cell type and internal solution, and when > two distances were measured per cell, a one-way repeated measures ANOVA was used, with activity-induced calcium influx as the repeated measure and distance from the soma as the factor. For comparing activity-induced dendritic calcium influx observed at > two distances form the soma across groups of OT-MCNs recorded with different internal solutions, or across different compartments of individual OT-MCNs, a two-way repeated measures ANOVA was used, with activity-induced calcium influx as the repeated measure, distance from the soma as one factor, and either internal solution or cellular compartment as the other. For all ANOVAs that involved repeated measures, Mauchly’s test of sphericity was used, and Greenhouse-Geisser corrections were applied if necessary. In cases where significant interaction between factors was noted, Holm-Sidak post-hoc tests were used to test for mean differences in measured values (e.g. of activity-induced calcium influx), across one factor (e.g. internal solution), at specific levels of the other (e.g. distance from the soma). In all cases, p-values ≤ alpha level of 0.05 were considered statistically significant. Error bars on all plots represent the SEM.

## Data Availability

All data generated or analysed during this study are included in the manuscript and supporting files. Source data files have been provided for all figures.

## Additional files

Transparent reporting form
