# Supplementary material for: Dendritic osmosensors modulate activity-induced calcium influx in oxytocinergic magnocellular neurons of the mouse PVN
Source: eLife. 2021 Jul 12;10:e63486. doi: 10.7554/eLife.63486 (PMC8457833; doi:10.7554/eLife.63486)
Supplement: Peer review history

# Decision letter

**DOI:** 10.7554/eLife.63486.sa1

**Affiliations:** Max Planck Florida Institute for Neuroscience United States; Tulane University United States

In the interests of transparency, eLife publishes the most substantive revision requests and the accompanying author responses.

**Acceptance summary:**

This study examined osmolarity-dependent dendritic signaling in oxytocin magnocellular neurosecretory cells (OT-MNCs). The authors show that repetitive depolarizations evoke larger calcium responses in proximal dendrites relative to distal dendrites. When these neurons were exposed to hyperosmotic stimuli, the distal calcium responses were found to be inhibited to a greater extent compared to proximal dendritic calcium responses. Propagation of glutamate evoked depolarizations from the dendrite towards the soma were also found to be reduced following increases in osmolarity. These effects of hyperosmotic stimuli are likely mediated by changes in membrane resistance of dendrites. A non-selective blocker of the channels, ruthenium red, blocked these effects of hyperosmolarity, indicating the non-selective cation channels (e.g. TRPV types) may be responsible. The finding is significant and addresses fundamental questions about MNC dendritic physiology.

**Decision letter after peer review:**

Thank you for submitting your article "Dendritic membrane resistance modulates activity-induced Ca^2+^ influx in oxytocinergic magnocellular neurons of mouse PVN" for consideration by *eLife*. Your article has been reviewed by 3 peer reviewers, and the evaluation has been overseen by a Reviewing Editor and John Huguenard as the Senior Editor. The following individuals involved in review of your submission have agreed to reveal their identity: Jeffrey Tasker (Reviewer #1); Rio Teruyama (Reviewer #2).

The reviewers have discussed the reviews with one another and the Reviewing Editor has drafted this decision to help you prepare a revised submission.

As the editors have judged that your manuscript is of interest, but as described below that additional experiments are required before it is published, we would like to draw your attention to changes in our revision policy that we have made in response to COVID-19 (https://elifesciences.org/articles/57162). First, because many researchers have temporarily lost access to the labs, we will give authors as much time as they need to submit revised manuscripts. We are also offering, if you choose, to post the manuscript to bioRxiv (if it is not already there) along with this decision letter and a formal designation that the manuscript is "in revision at *eLife*". Please let us know if you would like to pursue this option. (If your work is more suitable for medRxiv, you will need to post the preprint yourself, as the mechanisms for us to do so are still in development.)

Summary:

This study examined osmolarity-dependent dendritic signaling in oxytocin magnocellular neurosecretory cells (OT-MNCs). The authors show that repetitive depolarizations evoke larger calcium responses in proximal dendrites relative to distal dendrites. When these neurons were exposed to hyperosmotic stimuli, the distal calcium responses were found to be inhibited to a greater extent compared to proximal dendritic calcium responses. Propagation of glutamate evoked depolarizations from the dendrite towards the soma were also found to be reduced following increases in osmolarity. These effects of hyperosmotic stimuli are likely mediated by changes in membrane resistance of dendrites. A non-selective blocker of the channels, ruthenium red, blocked these effects of hyperosmolarity, indicating the non-selective cation channels (e.g. TRPV types) may be responsible.

All three reviewers agreed that the finding is potentially important and could address fundamental questions about MNC dendritic physiology. However, the reviewers identified a number of technical concerns, as summarized below. These concerns need to be addressed for further consideration.

Essential revisions:

1) The title and abstract are not exactly reflecting what this study is about. The title of the paper is "Dendritic membrane resistance modulates activity-induced Ca^2+^ influx in oxytocinergic magnocellular neurons of mouse PVN". However, dendritic membrane resistance is never actually measured. As such, a title that does not mention membrane resistance may be more appropriate. Also, the purpose and rationale of this study are not clearly communicated in the abstract and introduction. The implication to the regulation of soma-dendritic release of oxytocin, but not hyperosmotic responses, was mentioned in Introduction, while the entire Results and Discussion sections are about hyperosmotic stress.

2) Figure 3: The reviewers believe that stimulation paradigm is not physiological (neurons voltage-clamped at -70 mV with repetitive voltage steps to +50 mV for 5 ms). It is important to show that action potentials in the current clamp, instead of the +50mV voltage step in the voltage-clamp, can produce similar signals.

3) A major focus of the manuscript is on Ca^2+^ elevations in MNC dendrites. However, the authors have not performed the essential experiments to identify what the Ca^2+^ entry/release pathways are. It is important to show that Ca^2+^ is through voltage-gated Ca^2+^ channels for their main conclusions. In addition, it should also be established whether dendritic propagation is active or passive.

4) It is essential to report the effect of the osmotic stimulus alone on dendritic resting Ca^2+^, as this would affect the interpretation of the Ca^2+^ data.

5) Figure 8: What is the effect of RR on proximal EPSCs? This information is needed to interpret the effect of RR on distal EPSCs. It would be required to also test the effect of RR on the modulation of Ca^2+^ responses in distal dendrites to see their effects on the dendritic conductance.

Statistical handling:

Please provide the statistical methods (t-test, 2-way ANOVA with Hom-Sidak corrections, 2-way repeated-measures ANOVA, etc.) used for each measurement in the text or figure legend (not just in the method section). For repeated measures ANOVA, please indicate how measurements were repeated.

For the statistics of sex differences (Figure 2-1, 4-1 etc), it is required to use 3-way ANOVA to assess variability by cells, animals, and sex. The number of males and females used is not clear in some cases, but it appears that only 2 females and 2 males are used (Line 203-204). If this is the case, the statistical comparisons between males and females are not meaningful and should be removed.

[Editors' note: further revisions were suggested prior to acceptance, as described below.]

Thank you for resubmitting your work entitled "Osmosensitive dendritic ion channels modulate activity-induced calcium influx in oxytocinergic magnocellular neurons of the mouse PVN" for further consideration by *eLife*. Your revised article has been reviewed by 3 peer reviewers, and the evaluation has been overseen by a Reviewing Editor and John Huguenard as the Senior Editor.

The manuscript has been improved but there are some remaining issues that need to be addressed, as outlined below:

Essential revisions:

1. This study found were the effect of +15 mOsm ACSF and RR on Ca++ influx in the proximal and distal dendrites. We agree with the authors that the osmosensitive ion channels are most likely responsible for the changes in membrane resistance of dendrites; however, this study did not directly test the ion channels.

We suggest removing "ion channels" from the title, like "Osmolarity regulates activity-induced calcium influx in oxytocinergic magnocellular neurons of the mouse PVN".

2. If the hyperosmotic stimulation is modulating dendritic membrane resistance by opening channels, it would be expected to suppress the dendritic Ca response evoked by activation of the dendrites with glutamate application, if the glutamate-induced Ca response is caused by depolarization. The relative lack of effect of hyperosmotic stimulation on the dendritic Ca response to dendritic glutamate application suggests that the effect is not a simple modulation of membrane resistance.

In the last section of the Results (GABA_A_ receptor activation), since the voltage change was generated in the soma, it is not surprising that the distal dendritic response would be more attenuated than the proximal dendritic response with GABA_A_ receptors held open along the length of the dendrite. This doesn't appear to add anything new to the study, since the general increase in membrane conductance caused by tonic GAABAa receptor activation is expected to shunt the response and the shunting effect should be more pronounced further away from the stimulus. It seems that the main take-home message of the study is that dendrites express osmosensitive channels that attenuate the dendritic response and that are different from the osmosensitive channels expressed in the soma.

We suggest discussing this point.

3. As pointed out previously, it is surprising that the proximal dendritic response is not affected by the hyperosmotic stimulation, since the somatic response to current injection (i.e., spiking) is affected, ostensibly via TRPV modulation. Was there a change in the input resistance in the somatic recording in response to the hyperosmotic stimulation that underlies the change in spiking?

Please provide the data of input resistance, and discuss the point mentioned above.

4. In the Results section on distinct somatic vs. dendritic effects of osmotic stimulation (note: osmotic "stress" is still used throughout the manuscript: please fix this), it is implied that a novel finding of the study is that the osmosensor in the soma is a TRP channel. While this is discussed in the Discussion, no mention is made in the Results of the fact that Bourque et al. demonstrated previously that TRPV1 channels are responsible for the osmosensitivity of VP neurons, such that the current findings suggest that the same mechanism is at play in the somatic osmosensitivity of OT neurons. Presumably, the previous findings informed the experiment here to test for TRP channel involvement.

We suggest adding this point in the Result section to clarify the rationale of the experiment.

5. Relatedly, when introducing and interpreting the ruthenium red experiment in the Results, the Bourque findings of a TRPV dependence of the somatic osmosensitivity should be cited. This justifies your testing of RR to target TRP channels and eliminates the implication on lines 338-39 that the somatic sensor being a TRPV channel is a novel finding (although it is new to the OT neurons). Please clarify this point.

# Reply: Author response

**DOI:** 10.7554/eLife.63486.sa2

Essential revisions:

1) The title and abstract are not exactly reflecting what this study is about. The title of the paper is "Dendritic membrane resistance modulates activity-induced Ca^2+^ influx in oxytocinergic magnocellular neurons of mouse PVN". However, dendritic membrane resistance is never actually measured. As such, a title that does not mention membrane resistance may be more appropriate. Also, the purpose and rationale of this study are not clearly communicated in the abstract and introduction. The implication to the regulation of soma-dendritic release of oxytocin, but not hyperosmotic responses, was mentioned in Introduction, while the entire Results and Discussion sections are about hyperosmotic stress.

In response to this comment, we have changed the title to: “Osmosensitive dendritic ion channels modulate activity‐induced calcium influx in oxytocinergic magnocellular neurons of the mouse PVN”. This change both removes the term ‘dendritic membrane resistance’ from the title and draws more attention to the focus on osmosensitive mechanisms. That said, please note that experiments presented in Figure 9 demonstrate that activity‐induced calcium influx in OT‐MCN dendrites can also be similarly modulated by stimuli that do not change osmotic pressure. We believe that this is an important point in the overall scope of the manuscript. As such, text in the abstract has been updated to explicitly reference effects of both osmosensitive and non‐osmosensitive ion channels on activity‐induced calcium influx in OT‐MCN dendrites, and both types of results are further previewed in the Introduction.

2) Figure 3: The reviewers believe that stimulation paradigm is not physiological (neurons voltage-clamped at -70 mV with repetitive voltage steps to +50 mV for 5 ms). It is important to show that action potentials in the current clamp, instead of the +50mV voltage step in the voltage-clamp, can produce similar signals.

In response to this comment, we have completed a new experiment in current clamp that evaluates the effect of acute hyperosmotic challenge on activity‐induced calcium influx, in both proximal and distal dendrites of OT‐MCNs, using a train of 250 μs long suprathreshold current pulses, still delivered at 20 Hz for 2 sec, to evoke action potentials. The amplitude and kinetics of action potentials produced by this stimulus closely matched spontaneous action potentials observed in the same cells. Using this protocol, we found that acute hyperosmotic challenge reduced activity‐induced calcium influx in the proximal and distal dendrites by ‐13.3 ± 3.61% and ‐38.3 ± 3.92%, respectively. These results closely matched those observed with the original protocol (reduction of ‐14.3 ± 3.37% and ‐40.6 ± 4.23% in the proximal vs. distal dendrites, respectively). These new data are now presented in the Results section of the manuscript, and in more detail in Figure 4-figure supplement 3.

3) A major focus of the manuscript is on Ca^2+^ elevations in MNC dendrites. However, the authors have not performed the essential experiments to identify what the Ca^2+^ entry/release pathways are. It is important to show that Ca^2+^ is through voltage-gated Ca^2+^ channels for their main conclusions. In addition, it should also be established whether dendritic propagation is active or passive.

In response to the first point, we performed a new experiment that evaluates the effect of removing extracellular calcium on activity‐induced calcium influx, as observed in both proximal and distal dendrites of OT‐MCNs. Specifically, we found that transient (15‐minute) bath application of calcium free ACSF reduces activity‐induced calcium influx observed in the proximal and distal dendrites by 80.3 ± 3.5% and by 67.9 ± 3.3%, respectively, and that the activity‐induced calcium signal rapidly recovers when extracellular calcium is restored. These results confirm that influx of extracellular calcium is the primary driver of activity‐induced calcium influx measured in our experiments. Given that the stimulus is somatic depolarization of the patched neuron only, and that both AMPA and NMDA receptors are blocked, these results further support the conclusion that VGCCs are required, without eliminating a possible downstream contribution of calcium induced calcium release. These data are now presented in the Results section and are illustrated in Figure 3-figure supplement 1. Additional data generated with low calcium and high magnesium ACSF (0.25 mM and 10 mM, respectively, intended to directly block a subset of VGCCs) also strongly reduced activity‐induced calcium influx in proximal dendrites (by 56.9 ± 2.3%, n=2).

With respect to the second point, about whether dendritic propagation is active or passive, we expect it is primarily passive because there is substantial loss of signal with increasing distance from the soma in control conditions, which is not observed in cells filled with a cesium based internal, as presented in Figure 3. That said, to more directly evaluate the possibility of a minor role for voltage gated sodium channels in supporting voltage propagation in OT‐MCN dendrites, we have now performed an additional analysis of the data from TTX experiments presented in Figure 4. Specifically, we calculated the ratio of the activity-induced calcium signal in distal vs. proximal dendrites of OT‐MCNs both before and after bath application of TTX. The results indicate that TTX has no impact on this ratio (n=5, ratio = 0.52 ± 0.1 and 0.40 ± 0.1 before and after bath application of TTX, respectively, t=1.43, p=0.23, two‐sample paired t‐test). Based on this analysis, which has been added to the Results section of the manuscript, we can now more confidently conclude that voltage‐gated sodium channels do not appear to contribute meaningfully to voltage propagation in OT‐MCN dendrites.

4) It is essential to report the effect of the osmotic stimulus alone on dendritic resting Ca^2+^, as this would affect the interpretation of the Ca^2+^ data.

In response to this comment, we have now completed a more thorough analysis of the effect of acute hyperosmotic challenge on resting calcium, in both proximal and distal dendrites, throughout the manuscript. We find that on average, basal calcium levels (as indicated by resting F/F) changed by ≤ 6.1% in response to acute hyperosmotic challenge, and importantly, that small fluctuations in resting calcium were not correlated with observed effects of acute hyperosmotic challenge on activity‐induced calcium influx. This conclusion is now directly stated in the text of the results and is presented in greater detail in Figure 4-figure supplement 2.

5) Figure 8: What is the effect of RR on proximal EPSCs? This information is needed to interpret the effect of RR on distal EPSCs. It would be required to also test the effect of RR on the modulation of Ca^2+^ responses in distal dendrites to see their effects on the dendritic conductance.

In response to the later parts of this comment, we have completed a new experiment which reveals that pre‐treatment with RR does not alter the effect of acute hyperosmotic challenge on activity‐induced calcium influx, as observed in either the proximal or distal dendrites of OT‐MCNs. The results have been added to Figure 8, in place of the results of experiments with distally evoked EPSCs. The eEPSC experiment is still presented in Figure 8-figure supplement 1. Collectively, we now provide data indicating that pre-treatment with RR does not change the effect of acute hyperosmotic challenge on activity‐induced calcium influx in either proximal or distal dendrites, or the effect of acute hyperosmotic challenge on the amplitude of distally evoked EPSCs. We did not evaluate the ability of RR to modulate the effect of acute hyperosmotic challenge on proximally evoked EPSCs in part because we believe this question is better addressed by the new calcium imaging data now highlighted in Figure 8, and in part because even absent RR, the effect of acute hyperosmotic challenge on proximally evoked EPSCs is significantly smaller than that observed using distally evoked EPSCs, as reported in Figure 7.

Statistical handling:

Please provide the statistical methods (t-test, 2-way ANOVA with Hom-Sidak corrections, 2-way repeated-measures ANOVA, etc.) used for each measurement in the text or figure legend (not just in the method section). For repeated measures ANOVA, please indicate how measurements were repeated.

We now provide statistical methods used in line with each result in either the main text or figure legend, and we have significantly updated the methods section covering statistical analysis to more clearly indicate when regular vs. repeated measures ANOVAs were used. Factors for each analysis are now also more specifically highlighted, and when present, factors with repeated measures are also noted.

For the statistics of sex differences (Figure 2-1, 4-1 etc), it is required to use 3-way ANOVA to assess variability by cells, animals, and sex. The number of males and females used is not clear in some cases, but it appears that only 2 females and 2 males are used (Line 203-204). If this is the case, the statistical comparisons between males and females are not meaningful and should be removed.

In response to this comment, we have removed the two‐sample proportion test previously used to evaluate co‐localization of tdTomato and NP1 in PVN neurons of males vs. females. Although other datasets used to evaluate sex differences have more animals available (as noted in text and/or source data files), we do not believe animal is a particularly informative/viable factor for a 3‐way ANOVA because in many cases only a small number of cells could be recorded per animal. As such, we use two‐way ANOVA with sex and cell type or dendritic location as factors for these analyses in the manuscript. That said, in response to this comment we did run a 3‐way ANOVA using data presented in Figure 1‐1 and 4‐1, with sex, animal, and cell‐type or dendritic location as factors, and noted that populations means separated by animal were not significantly different in any dataset, and that there were no significant interactions between animal and any other factor.

[Editors' note: further revisions were suggested prior to acceptance, as described below.]

Essential revisions:

1. This study found were the effect of +15 mOsm ACSF and RR on Ca++ influx in the proximal and distal dendrites. We agree with the authors that the osmosensitive ion channels are most likely responsible for the changes in membrane resistance of dendrites; however, this study did not directly test the ion channels.

We suggest removing "ion channels" from the title, like "Osmolarity regulates activity-induced calcium influx in oxytocinergic magnocellular neurons of the mouse PVN".

In response to this comment, we have changed the title to: “Dendritic osmosensors regulate activity-induced calcium influx in oxytocinergic magnocellular neurons of the mouse PVN.” This change removes the term “ion channels”, as requested, but retains an important key word ‘dendritic’.

2. If the hyperosmotic stimulation is modulating dendritic membrane resistance by opening channels, it would be expected to suppress the dendritic Ca response evoked by activation of the dendrites with glutamate application, if the glutamate-induced Ca response is caused by depolarization. The relative lack of effect of hyperosmotic stimulation on the dendritic Ca response to dendritic glutamate application suggests that the effect is not a simple modulation of membrane resistance.

Figure 5 highlights that whether the stimulus is somatic current injection, or dendritic depolarization produced by local application of glutamate, inhibition by acute hyperosmotic challenge is only apparent / robust when the response is measured distal from the stimulus that produced it. These results effectively highlight the effect of acute hyperosmotic challenge on dendritic conductivity.

By contrast, the relative lack of effect of hyperosmotic stimulation on the dendritic Ca response to dendritic glutamate application (measured at the site of application) does not mean that there is no change in local dendritic membrane resistance at all. Rather, it indicates that depolarization produced by local application of glutamate still drives the local membrane potential to a voltage that is suprathreshold for opening of voltage gated calcium channels. Importantly, we expect that any effect of hyperosmotic challenge on dendritic Rm occurs not just at the site of glutamate application, but along the whole length of the dendrite. Thus, we expect even a small local change in dendritic Rm (that is subthreshold for altering local glutamate evoked calcium influx) can have an additive effect on signal propagation, leading to a clear effect on glutamate‐evoked current arriving at the soma.

In the last section of the Results (GABA_A_ receptor activation), since the voltage change was generated in the soma, it is not surprising that the distal dendritic response would be more attenuated than the proximal dendritic response with GABA_A_ receptors held open along the length of the dendrite. This doesn't appear to add anything new to the study, since the general increase in membrane conductance caused by tonic GAABAa receptor activation is expected to shunt the response and the shunting effect should be more pronounced further away from the stimulus. It seems that the main take-home message of the study is that dendrites express osmosensitive channels that attenuate the dendritic response and that are different from the osmosensitive channels expressed in the soma.

We suggest discussing this point.

If GABA_A_ receptors were purely somatic, then the ratio of the effect in the distal dendrite vs. the proximal dendrite would be expected to be the same before and after muscimol, even though there would be less activity‐induced calcium influx at the distal dendrite than at the proximal dendrite in both conditions. In contrast, we find that muscimol, like acute hyperosmotic challenge, reduces activity-induced calcium influx at the distal dendrites more strongly than it does at the proximal dendrites. In our opinion, this is an important result in part because it highlights a role for GABA_A_ receptors expressed along the length of the dendrite in modulating activity‐induced calcium influx in OT‐MCNs. To our knowledge, this has not been functionally demonstrated in hypothalamus before, or more importantly, in neurons capable of releasing peptide from their dendrites in a calcium‐ and activity‐dependent manner. That said, this result is also quite important in our view because it indicates that dynamic regulation of dendritic conductance is likely to underlie the relationship between action potential firing and dendritic release of peptide from OT‐MCNs all the time, and not just in conditions that involve changes in osmolarity. Indeed, we expect further studies may reveal a range of additional endogenously available signaling molecules that will act on dendrites to modulate activity‐induced calcium influx at sites of dendritic peptide release. It is our hope that learning more about the range of signaling molecules and effector systems that can impact this relationship will ultimately help inform new therapeutic strategies for manipulating endogenous oxytocin signaling in the CNS.

We agree that a main take‐home message of the study is that dendrites express osmosensitive channels that attenuate the dendritic response and that are different from the osmosensitive channels expressed in the soma. These points are covered directly in the discussion (see sections beginning on line 407 and 426).

3. As pointed out previously, it is surprising that the proximal dendritic response is not affected by the hyperosmotic stimulation, since the somatic response to current injection (i.e., spiking) is affected, ostensibly via TRPV modulation. Was there a change in the input resistance in the somatic recording in response to the hyperosmotic stimulation that underlies the change in spiking?

Please provide the data of input resistance, and discuss the point mentioned above.

In response to the first part of this comment, it is important to note that activity‐induced calcium influx as observed at the proximal dendrite is significantly reduced by acute hyperosmotic challenge (Δ Peak ΔF/F proximal: ‐14 ± 3.37%, n = 13, t = ‐4.2 p – 0.001, as reported on line 178). This result was also reproduced, although in revision 1 not explicitly highlighted as statistically significant, in Figure 4 —figure supplement 3, using a current clamp protocol (Δ Peak ΔF/F proximal: ‐13.3 ± 3.6% n = 7, t = ‐3.7, p = 0.01). In our view, these results are likely produced primarily by activation of dendritic osmosensors along the first ~25 μm of the dendrite, and the effect that has on signal propagation between the soma and the site of dendritic calcium imaging. We have updated the text to better highlight these observations (see in particular updated legends for Figure 4 and Figure 4 —figure supplement 3).

In response to the second part of this comment, we acknowledge that acute hyperosmotic challenge (at +15 mOsm) had no statistically significant effect on somatic membrane resistance as measured using a standard membrane test protocol in voltage clamp, despite having a modest but statistically significant effect on spontaneous action potential firing frequency as observed in current clamp (+1.39 ± 0.55 Hz, as reported in Figure 2C). In response to prior reviews, we noted that it is likely that this change in frequency could be produced by quite a small change in membrane potential. Given the high basal input resistance of OT‐MCNs (well over 1 GΩ on average), we expect the somatic osmosensitive current underlying the effect on spontaneous firing frequency, and thus any associated change in somatic Rm associated with gating of osmosensors, is also quite small. Finally, we noted that although not statistically significant, in raw terms, somatic membrane resistance did decrease in response to acute hyperosmotic challenge (by 78 MΩ), consistent with other data that suggest opening rather than closing of a somatic conductance, such as TRPV1.

In the current revision, to evaluate this issue more thoroughly, we have completed additional voltage clamp experiments where we measured somatic membrane resistance in OT‐MCNs during acute hyperosmotic challenge under identical conditions, and with an identical protocol, but without concurrent 2P imaging of the dendrites, ultimately increasing n value in this dataset from 21 to 38. The additional data had no impact on the average somatic membrane resistance measured either before or after acute hyperosmotic challenge (before: 1435 ± 187.5 MΩ vs. 1355 ± 109.8 MΩ, n=21, 38, t = 0.40, p = 0.69, unpaired t‐test, after: 1,357 ± 177.9 MΩ vs. 1256 ± 109.3 MΩ, n=21, 38, t = 0.52, p = 0.61, unpaired t‐test) and the updated dataset continued to indicate that acute hyperosmotic challenge has no statistically significant effect on somatic membrane resistance (before hyperosmotic challenge: 1355 ± 109.8 MΩ, after: 1256 ± 109.3 MΩ, n=38, t = 1.38, p=0.18, paired t‐test). In the original dataset, mean somatic membrane resistance as observed in response to acute hyperosmotic challenge decreased by 78 MΩ (5.4%), and in the updated dataset it decreased by 99 MΩ (7.3%). A power analysis conducted using population means and overall variance from the updated dataset (n=38) indicates that 159 replicates would be necessary to detect an effect of this magnitude with an α level of 0.05 and power of 0.80. Importantly, this analysis does not mean that a very small change in somatic membrane resistance is not occurring, it simply means that any effect that does exist is too small to observe with statistical significance given our available sample size and overall population variance. This core result has been updated in the manuscript on line 182.

In response to the final part of this comment, we are not able to quantify the effect of acute hyperosmotic challenge on somatic membrane resistance in the same cells where the effect on spontaneous firing frequency was evaluated because those cells were recorded in current clamp during the osmotic stimulus using a protocol designed to continuously monitor spontaneous firing frequency.

4. In the Results section on distinct somatic vs. dendritic effects of osmotic stimulation (note: osmotic "stress" is still used throughout the manuscript: please fix this), it is implied that a novel finding of the study is that the osmosensor in the soma is a TRP channel. While this is discussed in the Discussion, no mention is made in the Results of the fact that Bourque et al. demonstrated previously that TRPV1 channels are responsible for the osmosensitivity of VP neurons, such that the current findings suggest that the same mechanism is at play in the somatic osmosensitivity of OT neurons. Presumably, the previous findings informed the experiment here to test for TRP channel involvement.

We suggest adding this point in the Result section to clarify the rationale of the experiment.

We have modified this section of the results to more directly describe prior work implicating TRPV1 channels in osmosensation of vasopressinergic neurons.

All remaining references to osmotic ‘stress’ have been removed from this section, and throughout the manuscript.

5. Relatedly, when introducing and interpreting the ruthenium red experiment in the Results, the Bourque findings of a TRPV dependence of the somatic osmosensitivity should be cited. This justifies your testing of RR to target TRP channels and eliminates the implication on lines 338-39 that the somatic sensor being a TRPV channel is a novel finding (although it is new to the OT neurons). Please clarify this point.

Edits made in response to point 4 above also now more explicitly indicate that prior work by Bourque and colleagues on VP neurons informed the choice of RR for present experiments.
